# Multifunctional Ag/MoO_3_ hybrid nanofiller-enabled PVA/CMC nanocomposite films: complementary enhancement of structural, optical, electrical, dielectric, and antibacterial properties

**DOI:** 10.1039/d6ra09276d

**Published:** 2026-10-26

**Authors:** G. M. Asnag, Mohammed ALSaeedy, Ramzan Ullah, Mohamed M. Badran, Aziz A. Aziz, Syed Mansoor Ali, Mansour K. Gatasheh, ZabnAllah M. Alaizeri

**Affiliations:** a Department of Physics, Faculty of Science, Mansoura University Mansoura Egypt; b Department of Chemistry, “Giacomo Ciamician” University of Bologna 40126 Bologna BO Italy mohammedali.alsaeedy@kocaeli.edu.tr; c Kocaeli University, Faculty of Arts & Sciences, Department of Chemistry 41001 Umuttepe-Kocaeli Turkey; d Shanghai Ultra-Precision Optical Manufacturing Engineering Center, Department of Optical Science and Engineering, Fudan University Shanghai 200433 China; e Department of Pharmaceutics, College of Pharmacy, King Saud University Riyadh 11495 Saudi Arabia; f Department of Physics and Astronomy, College of Science, King Saud University Riyadh-11451 Saudi Arabia zalaizeri@ksu.edu.sa; g Department of Biochemistry, College of Science, King Saud University P.O. Box 2455 Riyadh 11451 Saudi Arabia

## Abstract

In this study, Ag/MoO_3_ hybrid nanofiller-modified PVA/CMC nanocomposite films were prepared using a solution-casting method. The effect of addition of Ag/MoO_3_ on the functional properties of PVA/CMC matrix has been systematically studied emphasising the role of hybrid nanofillers in enhancing material properties. Structural analyses confirmed the successful integration of crystalline Ag and MoO_3_ phases within the polymer network, while FTIR results revealed evidence of possible polymer–nanofiller interactions. UV-visible analysis indicated that the optical absorption was enhanced and the optical band-gap energy was decreased due to the formation of localised electronic states and the enhanced interfacial charge-transfer. The electrical studies showed an increase in AC conductivity with increasing concentration of Ag and the 4 wt% Ag/MoO_3_ nanocomposite showed the best charge transport behaviour. The dielectric and impedance analyses showed increased interfacial polarization, reduced bulk resistance, and non-Debye relaxation behaviour associated with heterogeneous Ag/MoO_3_–polymer interfaces. Furthermore, the antibacterial activity was evaluated against *Staphylococcus* spp. and *Klebsiella pneumoniae*, where the nanocomposite films exhibited concentration-dependent bacterial inhibition. The highest antibacterial activity against *Staphylococcus* spp. and *Klebsiella pneumoniae* was exhibited by the PVA/CMC composite containing 4 wt% Ag/MoO_3_, indicating the effective antimicrobial contribution of the hybrid nanofiller. The obtained results demonstrate that Ag/MoO_3_-modified PVA/CMC nanocomposites possess multifunctional optical, electrical, dielectric, and antibacterial characteristics, indicating potential for future applications in dielectric components, antimicrobial coatings, and polymer-based functional materials.

## Abstract 2

Ag/MoO_3_ hybrid nanofillers tailor the optical, electrical, dielectric and antibacterial properties of solution-cast PVA/CMC films. The 4 wt% composite gives the best overall performance, with a 2.52 eV indirect band gap.

## Graphical abstract

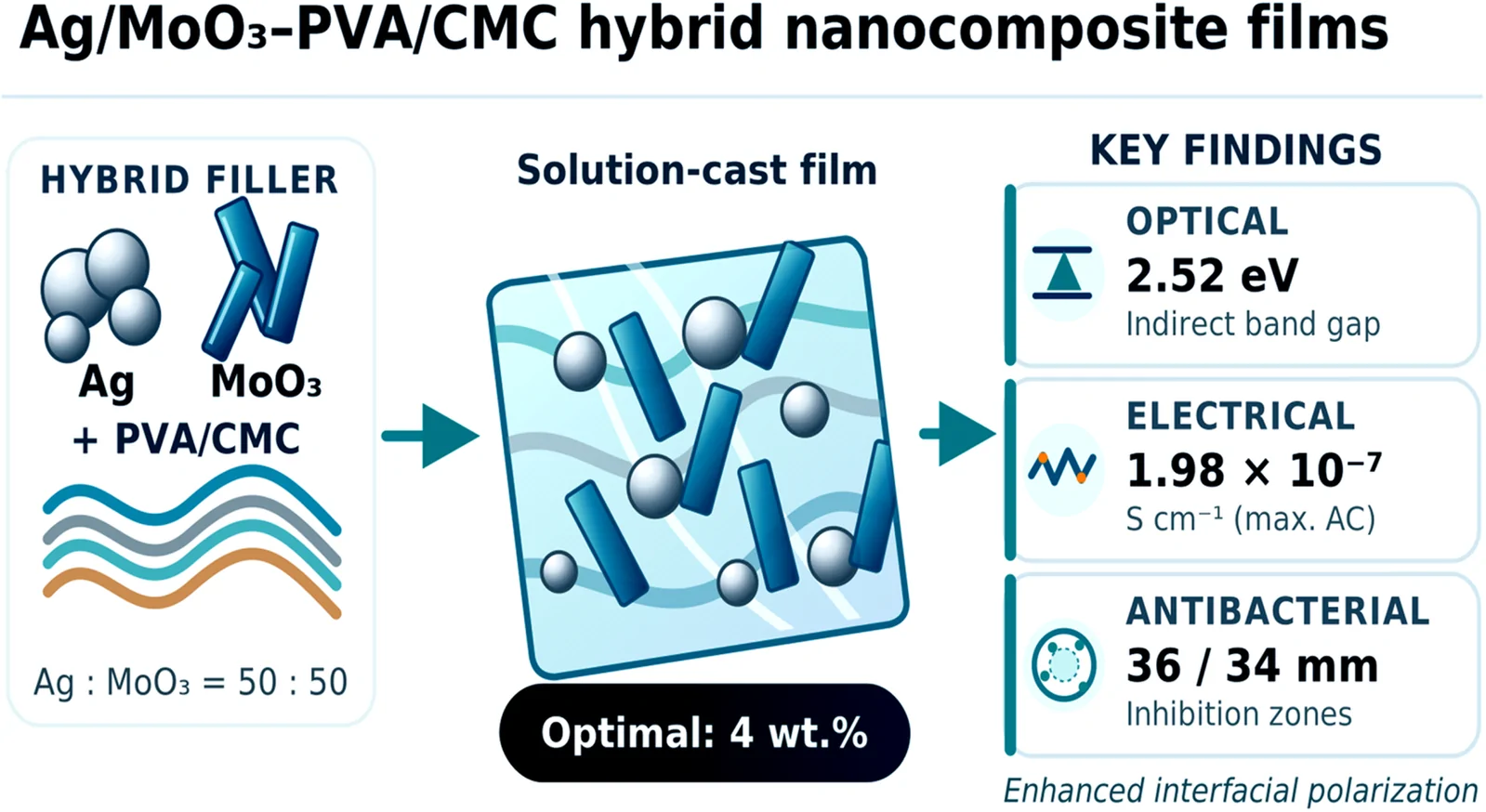

## 1 Introduction

The rapid development of wearable electronic devices, solid-state batteries, electrochemical storage systems, and multifunctional protective materials has led to the increasing demand for advanced materials with electrical functionality, lightweight, and environmental adaptability. Conventional inorganic materials usually show excellent electrical and optical performance. However, their inherent rigidity and limited processability hinder their application in flexible and deformable systems. Polymer-based materials and polymer nanocomposites have received much attention in research because of their structural versatility, chemical tunability and processing flexibility. The intrinsic advantages of the material properties permit their tailoring through molecular design, blending strategies for polymers, and the incorporation of functional nanofillers, thus opening vast opportunities for the development of advanced materials with tailored electrical and functional properties.^1–4^

Polymer nanocomposites are an efficient approach to improve the functional properties of polymer matrices, since nanoscale fillers can provide additional interfaces, modify polymer chain organization, and offer novel pathways for charge transport, optical interaction, and biological activity. The properties of these hybrid systems are strongly dependent on the type of filler, its morphology, concentration, quality of dispersion, and interaction between the nanomaterials and the polymer chains. Especially, the polymer nanocomposites with multifunctional properties comprising conductive metals and semiconductor oxides attracted a lot of attention as they can offer improved electrical, dielectric, optical and antimicrobial properties simultaneously.^5–7^

Among the polymer matrices, polymer matrices such as poly(vinyl alcohol) (PVA) and carboxymethyl cellulose (CMC) have gained much attention due to their excellent film-forming ability, hydrophilicity, and compatibility with inorganic nanomaterials.^4,5^ PVA is a semi-crystalline polymer with a large number of hydroxyl groups promoting hydrogen bonding and acting as active sites for interaction with nanoparticles. CMC is a polymer prepared from cellulose with hydroxyl and carboxymethyl functional groups, which can interact with PVA chains and incorporated nanofillers. The mixture of PVA–CMC forms a flexible polymer network with better structural stability while the functional groups are still available to take part in interfacial interactions.^5,7^

However, pristine PVA/CMC blend tend to exhibit low electrical conductivity and dielectric response because of the insulating behaviour of the polymeric matrix. The incorporation of nanoscale inorganic fillers offers an efficient route to overcome these limitations by generating additional charge-transfer pathways, altering polarisation behaviour and introducing localised electronic states.^5,7–12^

Previous works have demonstrated that the incorporation of functional nanofillers into PVA/CMC-based matrices has been shown to be an effective way to tune their structural, optical, electrical, dielectric and biological properties. Al-Muntaser *et al.* showed that the incorporation of α-MoO_3_ nanobelts in CMC/PVA composites produced significant modifications in crystallinity, optical energy, dielectric response, and ionic conductivity as a consequence of the enhanced interactions between the polymer and nanofiller and the interfacial polarisation.^5^ Likewise, Asnag *et al.* reported improved optical and dielectric features in MoO_3_-doped PVA/CMC nanocomposites due to the production of defect-related electronic states and modified charge transport pathways.^13^

Metallic nanoparticles, especially silver nanoparticles (Ag NPs), have also been widely explored as effective fillers for enhancing the functional attributes of polymer nanocomposites. Manne *et al.* prepared PVA/CMC/Ag nanocomposite films were fabricated using bio-origin silver nanoparticles, revealing significant improvements across multiple performance parameters, including morphology, thermal behavior, optical properties, electrical conductivity, and antibacterial efficiency due to the intrinsic antimicrobial nature of silver nanoparticles.^14^ Salim *et al.* studied solid polymer nanocomposites incorporated with Ag nanoparticles and found significant improvement in electrical conductivity and change in optical properties due to enhanced charge transport pathways and interaction between Ag nanoparticles and polymer chains.^8^

Similarly, Al-Marhaby *et al.* showed that Ag nanoparticles have a great effect on the crystallinity, optical response, dielectric behaviour and ionic conductivity of PVA-based polymer blends. The authors have shown that Ag nanoparticles are involved in the control of interfacial polarisation and electronic transitions.^9^

Beyond their electronic advantages, Ag nanoparticles exhibit broad-spectrum antibacterial activity against diverse bacterial species. Ag nanoparticles have been reported to exhibit antimicrobial activity through different mechanisms such as Ag^+^ ions release, interactions with bacterial cell membranes, disturbance of membrane permeability, generation of reactive oxygen species (ROS) and interaction with essential intracellular biomolecules.^15–18^ These properties have promoted the development of Ag-containing polymer nanocomposites to be used in antimicrobial coatings, biomedical materials, food packaging films and protective surfaces. According to Nasrallah and Ibrahim, the integration of silver-containing nanoparticles within PVA/CMC blend matrices improved their biological activity and modified their optical and dielectric responses. The study suggested that Ag-reinforced polymer systems can serve as promising platforms for multifunctional applications.^19^ These results demonstrate that Ag-based polymer nanocomposites can be used for electrical functionality and biological protection.

Molybdenum trioxide (MoO_3_) is one of the transition metal oxide semiconductors with unique electronic, optical and defect related properties. The incorporation of MoO_3_ modifies the mechanisms of charge transport and optical transitions of the matrices due to the presence of oxygen vacancies and surface active sites. Previous studies on MoO_3_/PVA/CMC systems have demonstrated significant enhancements in the modulation of the optical band-gap, dielectric response, electrical conductivity, and relaxation behaviour due to the interaction between the MoO_3_ nanostructures and the polymer groups.^5,12,20^ Additionally, MoO_3_-based nanomaterials have shown promising antibacterial activities *via* surface-mediated oxidative processes and active sites related to defects.^20^

The combination of Ag nanoparticles and MoO_3_ nanostructures offers an attractive strategy for designing multifunctional polymer nanocomposites because these two fillers possess complementary characteristics. Ag provides highly conductive metallic domains and strong antibacterial activity, and MoO_3_ exhibits semiconductor behaviour, defect-mediated electronic states and enhanced interfacial polarization. The interaction between Ag and MoO_3_ can form metal–semiconductor interfaces, which affect charge transfer, carrier localization, optical transitions, and surface activity. Thus, an Ag : MoO_3_ weight ratio of 50 : 50 was selected to balance the contribution of both phases and promote the multifunctional enhancement. Such hybrids can simultaneously improve electrical, dielectric, optical and antibacterial performances.^14,19–23^

Despite the advances, most of the previous investigations have been focused either on Ag-modified polymer systems or MoO_3_-containing polymer nanocomposites separately. The simultaneous incorporation of Ag NPs and MoO_3_ nanostructures into a PVA/CMC matrix is scarcely addressed, especially in relation to the correlation between the hybrid filler concentration, the interfacial interactions, the electrical/dielectric behaviour, and the antibacterial performance.^5,12^

Therefore, systematic investigation of Ag/MoO_3_ hybrid nanofillers within a PVA/CMC matrix is essential to understand their multifunctional behaviour and evaluate their potential for advanced applications such as dielectric components, antimicrobial coatings, and polymer-based functional materials, subject to further mechanical and device-level evaluation. Therefore, the present work highlights the synthesis of Ag/MoO_3_-reinforced PVA/CMC nanocomposite films through solution casting method. The effect of incorporation of hybrid nanofillers at different concentrations on the structural, optical, electrical, dielectric, impedance and antibacterial properties of the resultant nanocomposite films is systematically studied.

Structural modifications and polymer–nanofiller interactions are studied by XRD, FTIR, and TEM analyses. Optical transitions are studied by UV-visible spectroscopy and optical band-gap calculations. The electrical conductivity, dielectric behaviour, electric modulus and impedance characteristics are studied to understand charge transport and relaxation mechanisms. In addition, the antibacterial activity of the developed nanocomposites is studied against *Staphylococcus* spp. and *Klebsiella pneumoniae* to determine their potential as multifunctional antibacterial polymer materials.

## 2 Materials and experiments

### 2.1 Materials

PVA and CMC were selected as the blend matrix components for the preparation of PVA/CMC blend matrix. PVA powder (average molecular weight ≈ 14 kg mol^−1^) and CMC powder (average molecular weight ≈ 250 kg mol^−1^) were supplied by BDH Chemical Ltd, UK.

MoO_3_ nanobelts supplied by Alfa Aesar were utilized as the semiconductor filler in this study. The material possessed a purity of 99.5%, a molecular weight of 143.95 g mol^−1^, and a particle size smaller than 100 nm. Silver nitrate (AgNO_3_) and sodium borohydride (NaBH_4_) were obtained from Sigma-Aldrich (USA). Double-distilled deionized water served as the solvent throughout the experimental procedures.

### 2.2 Synthesis of Ag nanoparticles

Silver nanoparticles were produced using a chemical reduction approach, where silver nitrate (AgNO_3_) served as the metal ion source and sodium borohydride acted as the reducing agent. For the synthesis, 8 mL of 0.001 M AgNO_3_ aqueous solution was slowly introduced into 60 mL of freshly prepared 0.002 M NaBH_4_ solution under constant magnetic agitation at ambient temperature. The gradual addition of the precursor solution was carried out to facilitate controlled nucleation and uniform formation of Ag nanoparticles.

Prior to the reaction, the NaBH_4_ solution was cooled in an ice bath (≈0–5 °C) to control the reduction rate, suppress excessive nucleation and particle aggregation, and improve the stability of the resulting Ag nanoparticle colloid.

During the addition of AgNO_3_, the reaction mixture was maintained under vigorous magnetic stirring (≈530–550 rpm) to achieve uniform dispersion and efficient reduction of Ag^+^ ions by borohydride ions (BH_4_^−^). The reduction process resulted in the formation of silver atoms, followed by nucleation and growth of Ag nanoparticles. After complete addition of the AgNO_3_ solution, the suspension was stirred continuously for a further 5 min to complete the reduction reaction.

The formation of Ag nanoparticles was confirmed by gradual change in the colour of the reaction mixture from colourless to pale yellow to yellowish-brown, which is due to the surface plasmon resonance (SPR) phenomenon of nanoscale silver particles. The obtained Ag nanoparticle colloidal suspension was used for the subsequent preparation of Ag/MoO_3_ incorporated PVA/CMC nanocomposite films.

### 2.3 Fabrication of PVA/CMC–Ag/MoO_3_ nanocomposite films

PVA/CMC blend and Ag/MoO_3_-loaded PVA/CMC nanocomposite samples were prepared *via* the solution-casting method. Separate aqueous solutions of PVA and CMC were first obtained by dissolving the respective polymer powders in double-distilled deionized water at 65 °C and 55 °C, respectively, under continuous stirring for 6 h. The resulting solutions were then mixed at a PVA : CMC weight ratio of 70 : 30 and maintained under stirring at 60 ± 1 °C for 4 h to achieve complete blending and enhanced polymer chain interactions.

The obtained transparent and homogeneous blend solution of PVA/CMC was used as a polymer matrix for the preparation of pristine and Ag/MoO_3_-loaded nanocomposite films.

PVA/CMC–Ag/MoO_3_ nanocomposite films were fabricated by mixing appropriate amounts of Ag nanoparticles and MoO_3_ nanobelts in the ratio of 50 : 50 (by weight) dispersed in double distilled deionised water. The resulting hybrid nanofiller suspension was ultrasonically treated for 6 min to improve the dispersion of the filler and reduce agglomeration. Then, the well dispersed Ag/MoO_3_ nanofiller suspension was slowly added to the PVA/CMC blend solutions under magnetic stirring.

Nanocomposite solutions with total Ag/MoO_3_ hybrid nanofiller concentrations of 1.0, 2.0, 4.0 and 6.0 wt% relative to the PVA/CMC polymer blend were prepared, and the unfilled PVA/CMC film was used as the reference sample. The final solutions were stirred continuously until uniform dispersions were obtained and cast onto Petri dishes. After casting, the films were subjected to oven drying at a constant temperature of 40 ± 1 °C for 60 h. This controlled drying process enabled gradual solvent evaporation and promoted the development of uniform self-supported film structures.

### 2.4 Characterization methods

The fabricated PVA/CMC and Ag/MoO_3_/PVA/CMC nanocomposite films were characterized through various techniques to determine their structural, morphological, optical, electrical, dielectric, impedance, and antibacterial properties. TEM (JEM-1011, JEOL, Japan) was employed to investigate the morphology and size distribution of the synthesized Ag nanoparticles. The phase structure and crystallinity of Ag nanoparticles, MoO_3_ nanobelts, PVA/CMC films, and Ag/MoO_3_-reinforced nanocomposites were evaluated by XRD analysis (X'Pert PRO MRD, Malvern Panalytical) using Cu Kα radiation (*λ* = 1.5406 Å) within the 2*θ* range of 10–70°.

The functional groups characteristic and the potential interactions between PVA/CMC chains and Ag/MoO_3_ hybrid nanofillers were evaluated by FTIR (JASCO FTIR-430, Japan), in the range of 400 to 4000 cm^−1^.

The optical properties of the prepared films were studied by UV-visible spectrophotometer (JASCO V-630, Japan) in the range of 200–1000 nm.

Broadband dielectric spectroscopy system (Novocontrol Turnkey Concept 40) was used to measure the electrical and dielectric properties of the nanocomposite films at 303 K in the frequency range of 10^−1^ Hz–20 MHz. The frequency dependent dielectric constant, loss, AC conductivity, electric modulus and impedance response were studied to understand the charge transport, polarisation and relaxation mechanisms.

### 2.5 Assessment of antibacterial activity

The antibacterial performance of pristine PVA/CMC and Ag/MoO_3_-modified PVA/CMC films was evaluated using the agar diffusion method against *Staphylococcus aureus* (ATCC 29213, Gram-positive) and *Klebsiella pneumoniae* (ATCC 43816, Gram-negative) bacteria. Circular film samples with a diameter of 10 mm were placed on bacteria-inoculated nutrient agar plates and incubated at 37 °C for 24 h. The antibacterial activity was determined by measuring the inhibition zone diameter surrounding each sample.

The antibacterial activity was evaluated as a function of Ag/MoO_3_ hybrid nanofiller concentration (0.0–6.0 wt%). The formation of a clear zone of inhibition around the nanocomposite films was considered an indication of bacterial growth inhibition. All antibacterial measurements were performed in triplicate, and the inhibition zone diameters were calculated and reported as mean ± standard deviation.

## 3 Results and discussion

### 3.1 TEM analysis

TEM analysis was used to study the morphology and particle size distribution of the synthesised Ag nanoparticles as shown in Fig. 1. The TEM micrograph shows mainly quasi-spherical Ag nanoparticles with some variation in particle size and a slight tendency to agglomerate. The observed aggregation is a consequence of the high surface energy of nano-sized silver particles leading to attraction between adjacent nanoparticles. Partial aggregation can also be induced in the sample preparation and drying processes for TEM analysis.^24–26^

**Fig. 1:**
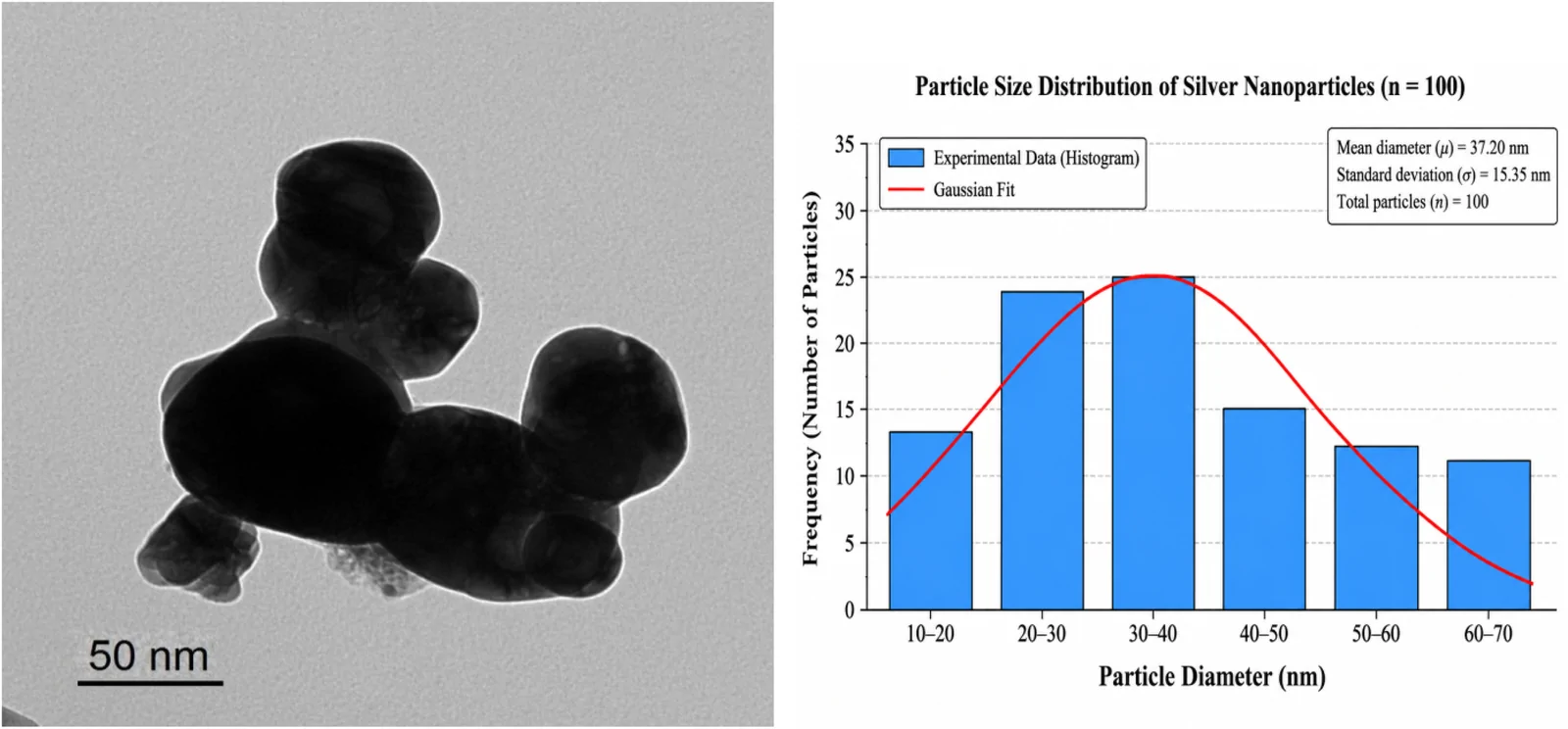
TEM micrograph and size distribution analysis of the synthesized silver nanoparticles (Ag NPs), showing quasi-spherical nanoparticle morphology with an average particle diameter of 37.20 ± 15.35 nm.

The histogram of the particle-size distribution of the synthesised Ag NPs is shown in Fig. 1 with diameters of 10–70 nm, in which most of the nanoparticles are distributed in the range of 20–40 nm. The fitting of the measured particle-size distribution gave an average particle diameter of 37.20 nm (with a standard deviation of 15.35 nm). The wide size distribution is in agreement with the variation of the nanoparticle sizes observed in the TEM images, and with the Ag nanoparticles prepared by chemical reduction as reported previously in the literature.^25,27^

The Ag nanoparticles exhibit a very strong dark contrast in the TEM images which is a consequence of the high atomic number of silver, which increases the electron scattering and results in higher mass-thickness contrast in TEM imaging.^28^ The darker regions seen in some areas are associated with the overlap of nanoparticles or small agglomerated clusters. TEM results confirmed the successful formation of nanoscale Ag nanoparticles with mainly quasi-spherical morphology and average particle size of 37.20 ± 15.35 nm. Such nanoscale features make the prepared Ag nanoparticles suitable to be included as a conductive and antibacterial ingredient in the Ag/MoO_3_-modified PVA/CMC nanocomposite matrix.

### 3.2 XRD analysis

The XRD patterns of the pristine PVA/CMC blend, synthesized Ag nanoparticles, MoO_3_ nanobelts, and Ag/MoO_3_-loaded PVA/CMC nanocomposite films are displayed in Fig. 2. XRD characterization was conducted to identify the crystalline phases of the incorporated nanofillers and evaluate the structural modifications occurring in the polymer matrix after hybrid filler addition.

**Fig. 2:**
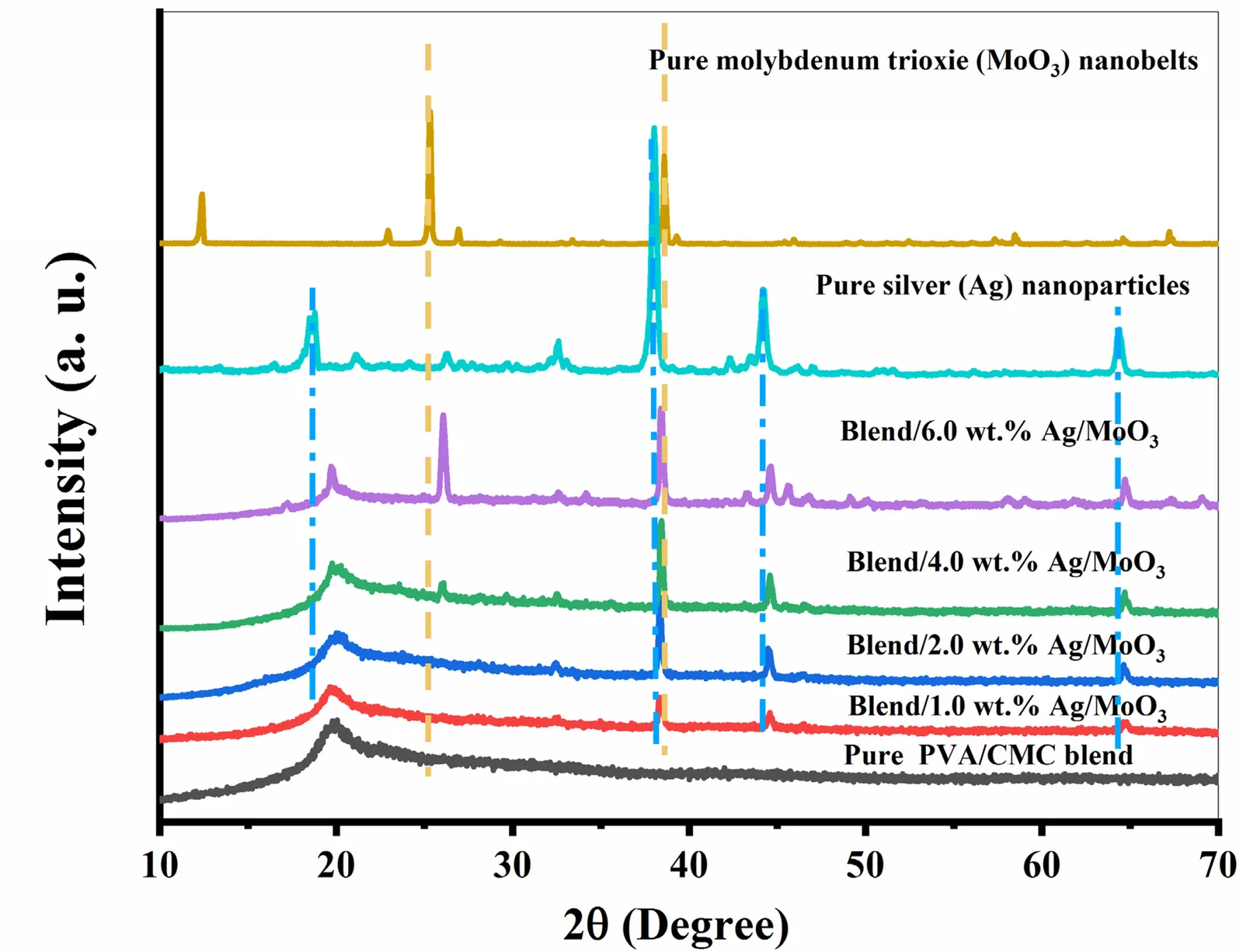
XRD patterns of pure Ag nanoparticles, pure MoO_3_ nanobelts, the pristine PVA/CMC blend, and Ag/MoO_3_-incorporated PVA/CMC nanocomposite films.

The diffraction profile of the prepared Ag nanoparticles exhibits characteristic peaks at approximately 2*θ* values of 38.0°, 44.3°, and 64.4°, corresponding to the (111), (200), and (220) crystal planes of the face-centered cubic (fcc) phase of metallic silver (JCPDS No. 04-0783).^29^ The presence of well-defined diffraction peaks confirms the successful formation of crystalline Ag nanoparticles. No other diffraction peaks were detected, indicating formation of phase-pure metallic Ag.

The diffraction pattern of MoO_3_ nanobelts shows characteristic reflections at approximately 2*θ* = 12.4°, 23.1°, 25.3°, 27.1°, 38.6°, and 39.3°, which can be indexed to the (100), (110), (040), (021), (111), and (151) planes of orthorhombic α-MoO_3_, respectively (JCPDS No. 05-0508).^12,30^ These diffraction peaks confirm the crystalline structure of the MoO_3_ nanobelts used as the semiconductor component of the hybrid nanofiller.

The XRD pattern of the pristine PVA/CMC blend exhibits a broad diffraction peak at approximately 2*θ* ≈ 19.9° indicating the semi-crystalline nature of the polymer matrix. The broad, low-intensity diffraction feature is suggestive of partially ordered polymer chains with limited crystalline domains. This behaviour is mainly attributed to the intermolecular hydrogen bonding interaction between the hydroxyl groups of PVA and the oxygen containing functional groups of CMC so as to affect the chain packing and crystallite formation.^12,20^

After the introduction of Ag/MoO_3_ hybrid nanofillers into the PVA/CMC matrix, the characteristic diffraction peaks of Ag and MoO_3_ phases are still observed, which indicates the successful incorporation and retention of the crystalline inorganic phases in the polymer network. No other peaks of secondary phases were found, which means that the hybrid filler components have preserved their structural identity during the composite preparation.

The peaks intensity of Ag and MoO_3_ was increased with the increase of Ag/MoO_3_ loading from 1.0 to 6.0 wt%, which is due to the higher concentration of crystalline nanofiller phases in the polymer matrix. In contrast, the intensity of the broad PVA/CMC diffraction halo is decreased after inclusion of nanofiller, indicating that the polymer chains are less ordered and less crystalline. The embedded Ag/MoO_3_ nanostructures can act as a barrier to the rearrangement of polymer chains by occupying the intermolecular space and limiting the formation of ordered crystalline regions. The crystallinity degree (*X*_cry_) of the polymer matrix was evaluated *via* the equation:^31^1
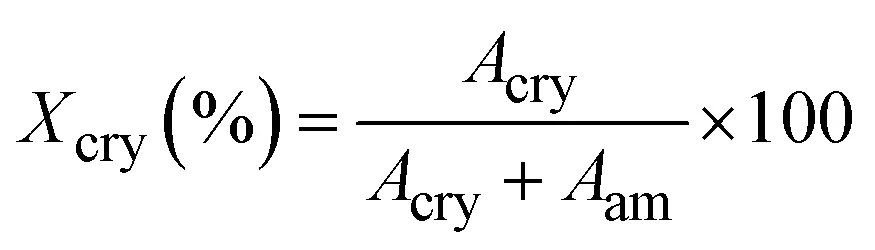
where *A*_cry_ represents the integrated area of the crystalline peaks and *A*_am_ corresponds to the integrated area of the amorphous halo. The calculated crystallinity degree (*X*_cry_) of pristine PVA/CMC blend matrix was found to be 36.6%. The crystallinity of the nanocomposite films gradually decreased to 27.1, 22.9 and 19.4% for the films containing 1, 2, and 4 wt% Ag/MoO_3_, respectively. The decrease in crystallinity may be associated with the addition of Ag/MoO_3_ nanofillers to the polymer network that hinder the re-arrangement of polymer chains, preventing the formation of ordered crystalline regions in the PVA/CMC matrix. The addition of the nanofillers increases the amorphous fraction by creating additional polymer–nanofiller interfaces and structural disorder. With increasing nanofiller concentration, the diffraction intensity of crystalline Ag and MoO_3_ phases increases, but these additional inorganic crystalline contributions do not compensate for the decrease of crystallinity of the polymer phase.^12,19^ The improved amorphous character of the composite network should promote the polymer-chain segmental mobility and offer novel pathways for charge-carrier transport, thereby contributing to the enhanced impedance and dielectric performance of Ag/MoO_3_-modified PVA/CMC nanocomposites.^12,32^

A slight increase in the calculated crystallinity degree to 19.7% was observed for the 6 wt% Ag/MoO_3_-loaded film. This behaviour may be related to the higher contribution of crystalline Ag and MoO_3_ phases, as evidenced *via* the increased intensity of their characteristic peaks, and possible local rearrangement of polymer chains at higher nanofiller loading. However, the crystallinity value remains substantially lower than that of the pristine PVA/CMC matrix, confirming that Ag/MoO_3_ incorporation primarily disrupts the original crystalline organization of the polymer network.

The red shift of the diffraction peaks of Ag and MoO_3_ after being incorporated into the PVA/CMC matrix implies that there are interfacial interactions between the nano-fillers and polymer chains. Such interactions can cause local structural stress and lattice distortion resulting in a slight modification of the interplanar distance of the crystalline phases. These interactions can induce local structural strain and modify the interplanar spacing of the crystalline nanofiller phases. The change in the interplanar distance was calculated by Bragg's equation:^33^

2 2*d* sin *θ* = *nλ*

where *d* represents the interplanar spacing, and *λ* is the X-ray wavelength. The observed shift of the peak position of Ag and MoO_3_ diffraction reflections after their incorporation into the PVA/CMC matrix can be explained by interfacial interaction between the nanofiller surfaces and PVA/CMC functional groups.^5,34^ These structural changes indicate the presence of effective polymer–nanofiller interactions that are responsible for the enhancement of the multifunctional properties of the Ag/MoO_3_-modified PVA/CMC nanocomposite films.

In summary, the XRD results confirm the successful formation of Ag/MoO_3_ hybrid PVA/CMC nanocomposite films, in which the crystalline Ag and α-MoO_3_ phases are preserved within the semi-crystalline polymer matrix. The structural modifications induced by the hybrid nanofillers, including changes in polymer-chain ordering, crystallinity, and polymer–filler interfacial interactions, play an important role in influencing the optical, electrical, dielectric, and antibacterial performance of the nanocomposite films. Furthermore, the interfaces formed between Ag nanoparticles, MoO_3_ nanobelts, and PVA/CMC chains provide active regions for charge accumulation and interfacial polarization, thereby affecting the electrical transport and dielectric response of the developed nanocomposite films.

### 3.3 FTIR analysis

Fig. 3 presents the FTIR spectra of neat PVA/CMC blend and PVA/CMC nanocomposite films with 1.0, 2.0, 4.0, and 6.0 wt% of Ag/MoO_3_ hybrid nanofillers. The spectra after incorporating Ag/MoO_3_ still show the main characteristic absorption bands of PVA/CMC matrix, which means the main chemical structure of the polymer blend is kept. However, upon the addition of nanofiller, changes in band position, width and relative intensity are observed suggesting a modification of the local chemical environment of the polymer functional groups.

**Fig. 3:**
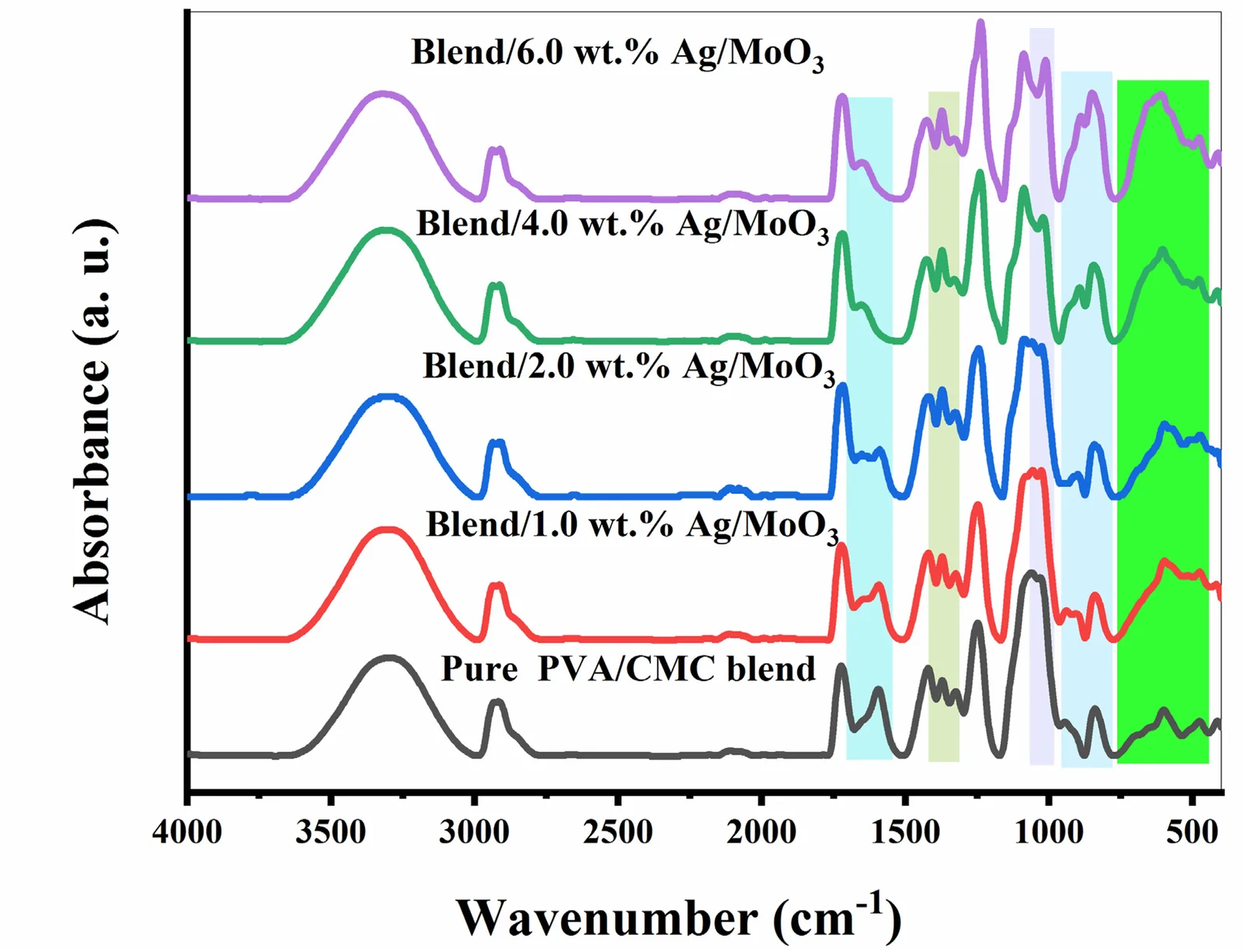
FTIR spectra of the pristine PVA/CMC blend, and Ag/MoO_3_-incorporated PVA/CMC nanocomposite films.

The wide absorption band observed at 3600–3050 cm^−1^ is mainly due to the O–H stretching vibrations of the hydroxyl groups of both PVA and CMC. This broad band shape is characteristic of the extensive hydrogen-bonding network in PVA/CMC blend.^35,36^ After incorporation of Ag/MoO_3_, changes in the position and width of the O–H band are observed. These variations are indicative of disturbance of the original hydrogen-bonding environment and are consistent with interactions between hydroxyl-containing polymer chains and the oxygen-rich surface of MoO_3_. These interfacial interactions can change the strength and distribution of hydrogen bonds in the polymer network.^12,20^

The absorption band at 2926 cm^−1^ is assigned to C–H stretching vibrations, mainly arising from the CH_2_ groups of the PVA backbone. The band observed at 1726 cm^−1^ is attributed to the C=O stretching vibration of residual acetate groups associated with partially hydrolysed PVA. In addition, the prominent absorption band at 1593 cm^−1^ was related to the asymmetric stretching vibration of carboxylate (COO^−^) groups present in CMC.^35,36^ These oxygen-containing functional groups can serve as active interaction sites for the incorporated Ag/MoO_3_ hybrid nanofillers, thereby contributing to polymer–nanofiller interfacial interactions.

The absorption band at 1422 cm^−1^ is attributed to overlapping CH_2_ bending vibrations and symmetric stretching of carboxylate (COO^−^) groups.^12^ The band at 1371 cm^−1^ is mainly associated with C–H bending vibrations. The feature observed at approximately 1325 cm^−1^ can be assigned to bending and wagging modes involving the polymer backbone and hydroxyl-containing groups. In addition, the two bands observed at 1251 and 1063 cm^−1^ are predominantly attributed to C–O and C–O–C stretching vibrations, respectively, which are characteristic of the PVA/CMC polymer matrix.^34–37^

Following Ag/MoO_3_ incorporation, loading-dependent changes are evident particularly in the O–H, C=O/COO^−^, and C–O/C–O–C regions. These variations indicate that hydroxyl, carboxylate, and ether-containing groups participate in interactions at the polymer–nanofiller interface. The oxygen-containing functional groups of PVA and CMC may interact with surface sites of MoO_3_ through hydrogen bonding, electrostatic attraction, and coordination-type interactions. Local environments may also be modified by interfacial interactions between Ag nanoparticles and neighbouring polymer functional groups. Thus, the spectral variations observed are more consistent with the modification of the inter- and intramolecular interactions than with the breaking of the polymer backbone.^35–37^

Significantly, no new separated absorptions related to new organic functional groups emerge upon the addition of Ag/MoO_3_. This observation indicates that the addition of hybrid nanofillers does not induce significant chemical degradation or large chemical modification of the PVA/CMC backbone. Rather, the FTIR results indicate that the interaction between polymer matrix and Ag/MoO_3_ is mainly characterised by the variations of hydrogen bonding and other interfacial interactions. However, FTIR cannot completely eliminate the formation of certain chemical bonds at the surface of nanofiller.^31,37^

Overall, the FTIR spectra suggest that Ag/MoO_3_ incorporation changes the local bonding environment of PVA/CMC without significantly affecting the characteristic chemical structure of the matrix. The variations in the O–H, COO^−^, C=O, and C–O/C–O–C regions indicate possible polymer–nanofiller interfacial interactions. These interactions are in agreement with the structural changes observed by XRD and may be responsible for the subsequent changes in the optical, dielectric and charge-transport behaviour of the Ag/MoO_3_-modified PVA/CMC nanocomposite films.

### 3.4 UV-visible analysis

The optical absorption behaviour of the pristine PVA/CMC matrix and the Ag/MoO_3_-modified PVA/CMC nanocomposite films was investigated using UV-visible spectroscopy, as shown in Fig. 4. The spectra provide information on the changes in the electronic structure of the blend following incorporation of Ag nanoparticles and MoO_3_ nanobelts, as well as on the contribution of polymer–nanofiller interfacial interactions to the optical response.

**Fig. 4:**
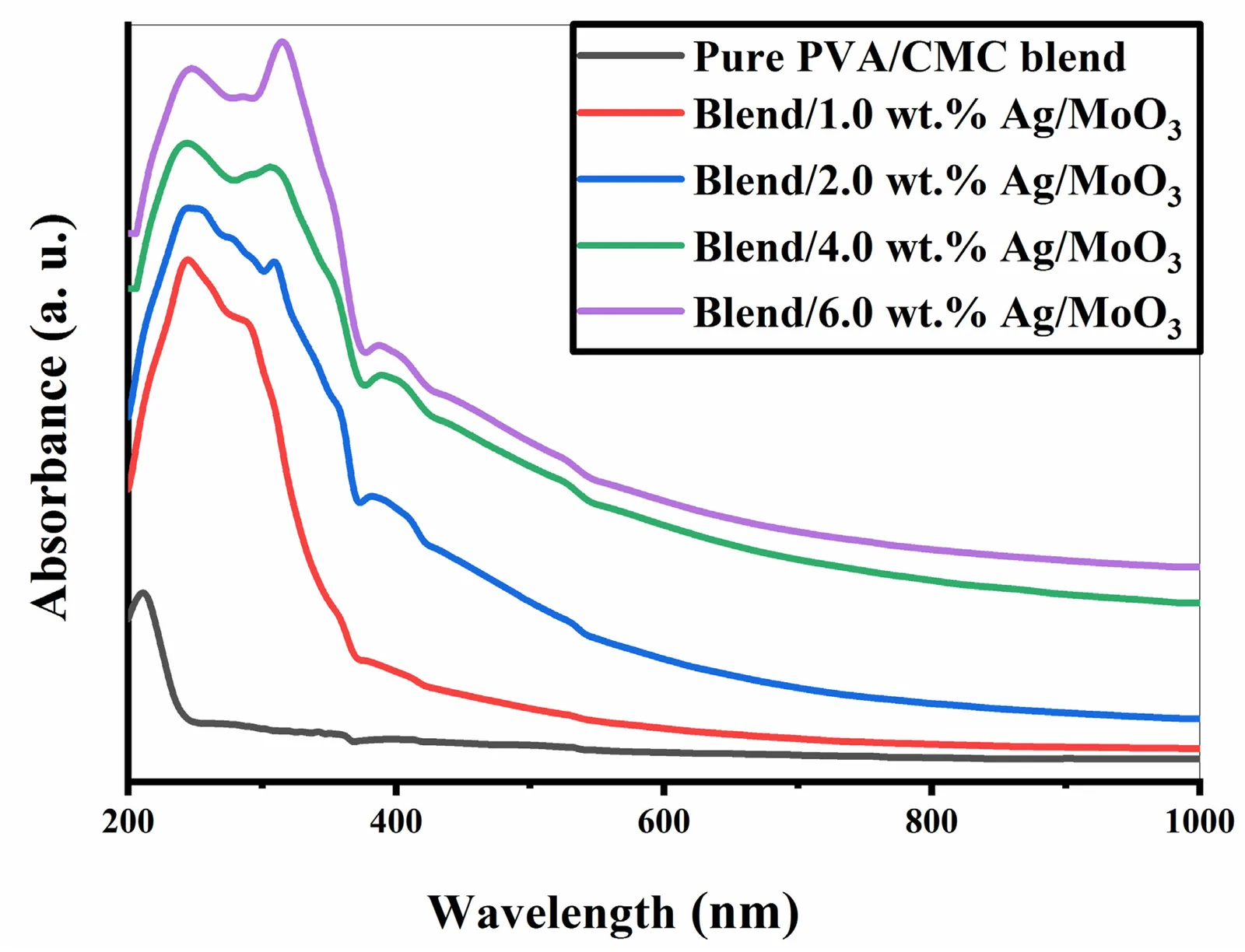
UV-visible absorbance spectra of the pristine PVA/CMC blend and Ag/MoO_3_-incorporated PVA/CMC nanocomposite films.

The pristine blend spectrum displays strong absorption in the ultraviolet part, with a maximum at 211 nm. This absorption is associated mainly with electronic transitions involving oxygen-containing functional groups in the PVA/CMC matrix. PVA contains abundant hydroxyl groups together with a small amount of residual acetate or carbonyl groups, whereas CMC contains hydroxyl, ether and carboxylate functionalities. Consequently, the absorption in this region may include contributions from transitions associated with these oxygen-containing groups and carbonyl-related chromophores.^12,19,38^ The strong absorption in the deep-UV region is consistent with the relatively wide optical band gap of the pristine polymer blend.

The incorporation of Ag/MoO_3_ hybrid nanofillers causes a clear modification of the UV-visible spectra. With increasing nanofiller content, the principal absorption feature shifts progressively towards longer wavelengths, from 211 nm for the pristine PVA/CMC film to about 247 nm for the film containing 6.0 wt% Ag/MoO_3_. This bathochromic shift indicates that less energy is needed for optical excitation and it is indicative of changes in the local electronic environment of the matrix. This behaviour can be attributed to polymer–nanofiller interactions, increased structural disorder and the formation of localised electronic states at the PVA/CMC–Ag/MoO_3_ interfaces.^12,38^

An additional absorption feature appears around 317 nm after Ag/MoO_3_ incorporation, and its intensity increases with increasing nanofiller precentage. This band can mainly be associated with the optical response of the MoO_3_ component. The absorption behaviour of MoO_3_ originates from electronic transitions between oxygen-derived valence band states and molybdenum-derived conduction band states, commonly described as O 2p → Mo 4d charge-transfer transitions.^30,39^ Furthermore, oxygen vacancies, defect-related states, and interfacial electronic states formed at the MoO_3_–polymer boundary can introduce intermediate energy levels, leading to broadening and enhancement of this absorption region.^30,40^

The absorption band at about 389 nm corresponds to localized surface plasmon resonance (LSPR) of Ag NPs which indicates the optical contribution of metallic silver in the PVA/CMC–Ag/MoO_3_ nanocomposite system. This plasmonic absorption is due to the collective oscillation of conduction electrons on the surface of Ag nanoparticles under incident radiation. The optical response of the Ag-LSPR band is determined by the physical properties of the Ag nanoparticles such as the size, shape and aggregation state as well as the dielectric interactions with the surrounding PVA/CMC matrix and the MoO_3_ nanostructures.^29,41^ The presence of this band indicates the contribution of silver to the optical response of the hybrid nanocomposite.

The gradual increase of absorption intensity with the increase of Ag/MoO_3_ loading from 1.0 to 6.0 wt% indicates an increased interaction of incident photons with the nanocomposite structure. The increasing nanofiller concentration leads to the increase of the number of polymer–nanoparticle interfaces, which serve as an active region for optical transitions, charge localization and interfacial polarization. The addition of nanoparticles altered the electronic structure of the host polymer by forming localized states^19,38^ and the PVA/CMC based nanocomposites with inorganic fillers also showed similar increase in optical absorption as reported.^19,38^

The improved optical response is primarily attributed to the semiconducting nature of MoO_3_, which can generate defect-related electronic states in the forbidden energy region of the polymer matrix.^30,42^ These localized states provide intermediate energy levels between the valence and conduction bands helping electronic excitation. Meanwhile, Ag nanoparticles contribute through their plasmonic response and their ability to modify the local electromagnetic environment of hybrid system.

Furthermore, the extended absorption tail toward the visible region after Ag/MoO_3_ incorporation indicates the increase of structural disorder and formation of additional localized states within the polymer network. These states may originate from the polymer–nanoparticle interactions, defect formation, and the interfacial electronic coupling between Ag, MoO_3_, and PVA/CMC chains. Such modifications are generally related to the reduction of optical band-gap energy and the enhancement of charge-carrier transport properties in polymer nanocomposite systems.^38,42^

Overall, the continuous enhancement in UV-visible absorption confirmed the successful incorporation of Ag/MoO_3_ hybrid nanofillers into PVA/CMC blend and modification of the matrix electronic structure. The observed red shift of the absorption edge (211–247 nm), MoO_3_ related absorption band (∼317 nm), Ag-LSPR peak (∼389 nm) and increased absorbance intensity suggest strong filler–matrix interactions. These results confirming that the incorporation of the nanofiller modifies the local structure and functional environment of the composite films.

### 3.5 Optical band gap analysis

The optical band-gap energies of the nanocomposite samples were calculated using Tauc relation as:^12^

3 (*αhν*)^*n*^ = *A*(*hν* − *E*_g_)

Here, *α* is the absorption coefficient, *hν* is the photon energy, *A* is a constant related to transition probability, *E*_g_ is the optical band-gap energy, and *n* represents the transition type.

Both direct and indirect transition models were considered to evaluate the possible electronic transition mechanisms in the Ag/MoO_3_-modified PVA/CMC system. The optical transitions were analyzed using Tauc plots of (*αhν*)^2^*versus hν* for direct transitions and (*αhν*)^1/2^*versus hν* for indirect transitions (Fig. 5a and b). The corresponding optical band-gap energies were determined by extrapolating the linear regions of the plots to the photon energy axis, and the obtained *E*_g_ values are listed in Table 1.

**Fig. 5:**
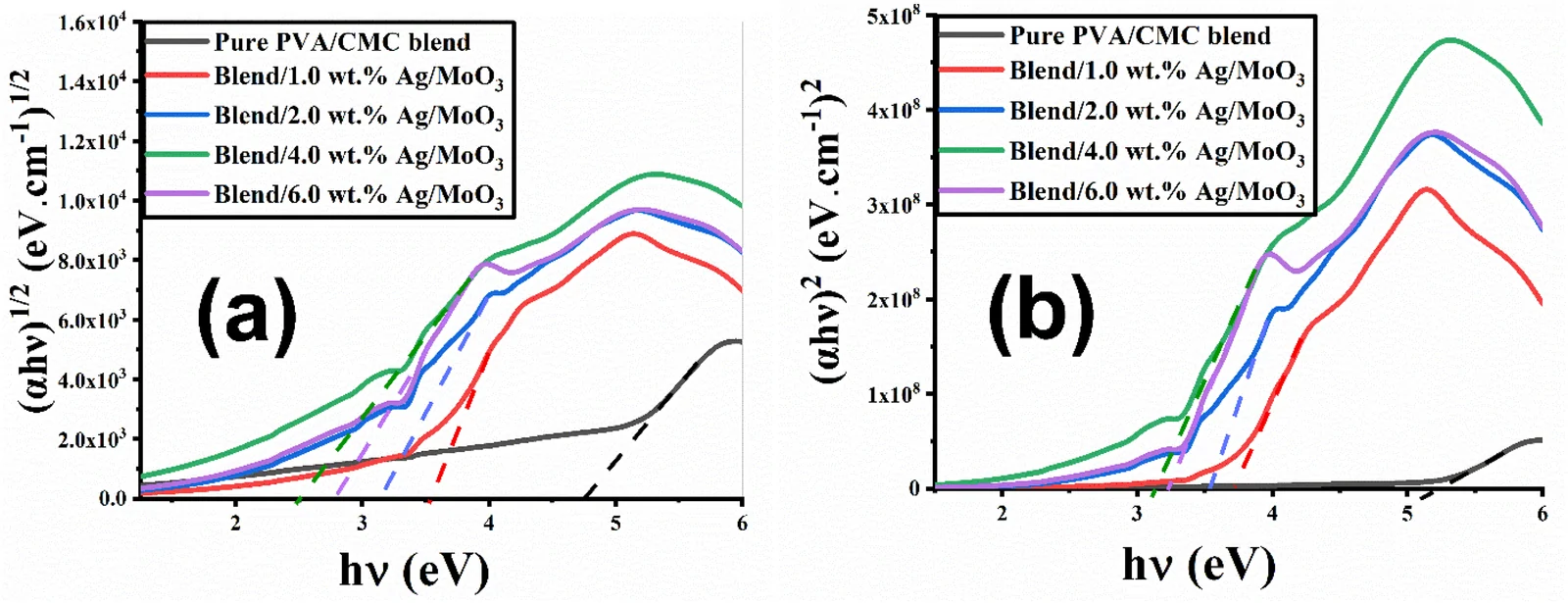
Tauc plots of PVA/CMC–Ag/MoO_3_ nanocomposite films: (a) (*αhν*)^1/2^ against (*hν*) for determining the indirect optical gap and (b) (*αhν*)^2^ against (*hν*) for determining the direct optical gap.

**Table 1:** Degree of crystallinity (*X*_cry_), indirect optical band-gap energy (*E*_gi_), and direct optical band-gap energy (*E*_gd_) of PVA/CMC–Ag/MoO_3_ nanocomposite films

Ag/MoO_3_ content (wt%)	*X*_cry_ (%)	*E*_gi_ (eV)	*E*_gd_ (eV)
0.0	36.6	4.78	5.22
1.0	27.1	3.54	3.73
2.0	22.9	3.13	3.54
4.0	19.4	2.52	3.14
6.0	19.7	2.76	3.23

The pristine PVA/CMC blend showed indirect and direct optical band-gaps of 4.78 and 5.22 eV, respectively. The band gaps are relatively large because of the insulating character of the matrix and the limited availability of localized electronic states for optical excitation. In the absence of inorganic nanofillers, electronic transitions require more photon energy, as the polymer network has fewer defect-related states in the forbidden energy region.^7,43,44^

Ag/MoO_3_ hybrid nanofiller incorporation significantly reduced the optical band-gap energies of the PVA/CMC films. The indirect band gap decreased from 4.78 eV for pristine PVA/CMC to 3.54, 3.13, and 2.52 eV, while the direct band gap decreased from 5.22 eV to 3.73, 3.54, and 3.14 eV for 1.0, 2.0, and 4.0 wt% Ag/MoO_3_ composites, respectively. The observed band-gap narrowing is attributed to the formation of additional electronic states and modification of the polymer–nanofiller interaction environment.

The reduction of band-gap energy could be attributed to a joint effect of Ag nanoparticles and MoO_3_ nanostructures in terms of formation of localized electronic states, defect-related levels and modified polymer–nanofiller interfaces. As a semiconductor oxide, MoO_3_ can introduce defect-related states associated with oxygen vacancies and localized electronic levels, and Ag nanoparticles contribute additional electronic interactions and promote charge-transfer processes at the heterogeneous interfaces. In addition, the surface-active sites of MoO_3_ may interact with the –OH groups present in PVA and the oxygen-containing groups of CMC. These interfacial interactions enhance the compatibility between the polymer matrix and nanofiller, leading to modifications in the electronic characteristics of the hybrid composite structure.^5,12,45^

The observed gradual decrease in band-gap energy up to 4 wt% Ag/MoO_3_ loading was related to the generations of localized electronic states and improved polymer–nanofiller interactions. These interfacial states promote electronic excitation by reducing the transition energy.^5,12,38^ The decrease in optical band gap due to localised states and structural modification has been reported for the Ag and MoO_3_ based PVA/CMC nanocomposites as well.^7,43,46^

Further increasing the Ag/MoO_3_ concentration to 6 wt% results in a small increase in both indirect and direct band-gap energies. The indirect and direct band gap values are enhanced to 2.76 eV and 3.23 eV respectively. This behaviour indicates that the excessive nanofiller loading may reduce the effectiveness of electronic interaction between the filler and polymer matrix. At higher concentration, possible aggregation of the nanofillers may reduce the available interfacial area, and influence the uniform distribution of localized electronic states. The aggregated nanoparticles may also break the continuous charge transfer pathways and lower the efficiency of electronic excitation in the composite structure.^8,47^

The optimum optical response is obtained at 4 wt% Ag/MoO_3_, where the balance between the nanofiller dispersion, formation of defect states and the polymer–nanofiller interaction is achieved. The lowest band gap energy at this concentration signifies improved optical activity and is consistent with the improved impedance and dielectric responses observed for the same composition.^12,48^

Overall, the Tauc analysis reveals that Ag/MoO_3_ hybrid nanofillers can be used to effectively tune the optical properties of the PVA/CMC matrix by modulating the electronic structure and producing additional localized level in the forbidden energy bands. The controlled reduction of the optical band-gap energy emphasizes the potential of these nanocomposite films in optoelectronic devices, UV-shielding materials, dielectric components and other polymer-based functional applications.

### 3.6 AC electrical conductivity analysis

Fig. 6 presents the AC conductivity (*σ*_ac_) of PVA/CMC and Ag/MoO_3_ nanocomposite films. The conductivity of all samples increases with frequency, which can be attributed to improved charge-carrier mobility and hopping conduction through localized states under an alternating field.^5,8^ The pristine PVA/CMC matrix shows the lowest conductivity due to its insulating polymeric nature and restricted charge-carrier density.^12^

**Fig. 6:**
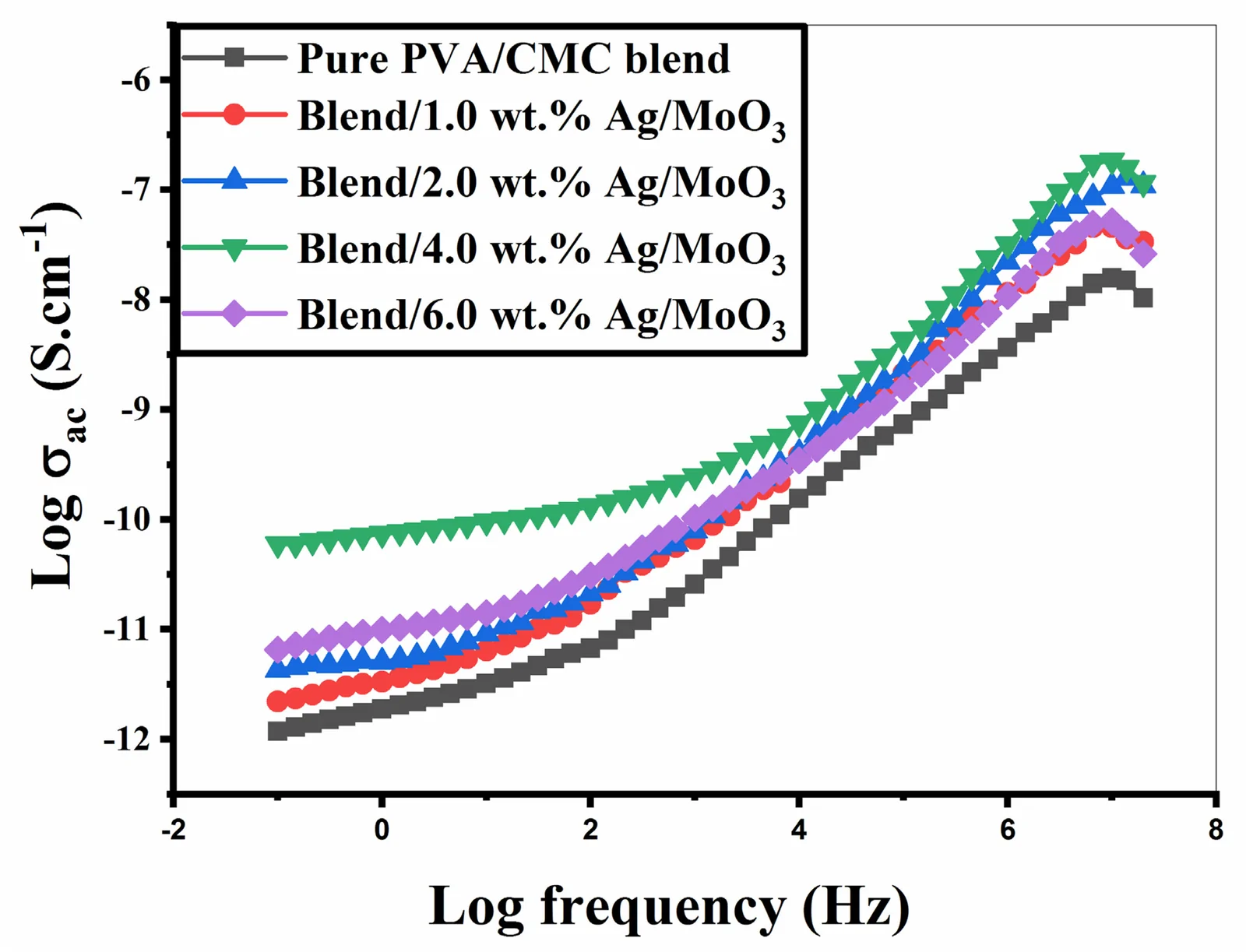
AC electrical conductivity (*σ*_ac_) of pristine PVA/CMC and Ag/MoO_3_-modified PVA/CMC nanocomposite films.

The addition of Ag/MoO_3_ hybrid nanofillers leads to a significant increase in conductivity, suggesting the role of inorganic nanophases in charge transport. The metallic Ag nanoparticles may provide conductive domains while MoO_3_ contributes localized electronic states associated with the semiconductor nature of MoO_3_ facilitating the carrier hopping through the polymer network.^7,12,19^

The increase in the values of *σ*_ac_ is more pronounced with the increase in the amount of Ag/MoO_3_ up to 4 wt%. Of all the composites, the 4 wt% Ag/MoO_3_ composite exhibits the highest values of *σ*_ac_ over the entire frequency range which could be ascribed to the production of an effective interconnected interfacial network among Ag, MoO_3_ and PVA/CMC chains. These heterogeneous interfaces are active regions for charge accumulation and facilitate charge-carrier transport *via* localized hopping mechanisms. The enhanced conductivity at this composition is in agreement with the minimum optical band-gap energy exhibited by the 4 wt% sample indicating the production of better electronic states and charge transfer capability.^12,38^

While higher loading of the nanofiller generally increases the conductivity, a slight decrease in the conductivity is obtained for the 6 wt% Ag/MoO_3_ composite in comparison to the 4 wt% composite. This reduction may be due to excessive nanofiller interactions and possible aggregation at higher loading, which could reduce the effective interfacial area and disturb the uniformity of conductive paths. A similar concentration dependent behaviour has been observed for polymer nanocomposites where the charge mobility can be reduced at higher filler loading due to aggregation effects.^47^

The AC conductivity behaviour of the Ag/MoO_3_ nanocomposite films was analysed by Jonscher's universal power law:^48^

4 *σ*_ac_ = *σ*_dc_ + *Aω*^*s*^

where *σ*_dc_ represents the frequency-independent conductivity, *A* is a constant, *ω* is the angular frequency, and *s* is the frequency exponent. The maximum AC conductivity values and Jonscher power-law parameters obtained for the prepared films are listed in Table 2.

**Table 2:** AC conductivity parameters of PVA/CMC–Ag/MoO_3_ nanocomposite films obtained from Jonscher's power-law analysis

Concentration of Ag/MoO_3_ (wt%)	Maximum *σ*_ac_ (S cm^−1^)	*σ*_dc_ (S cm^−1^)	Frequency exponent (*s*)
0.0	1.67 × 10^−8^	9.77 × 10^−13^	0.60
1.0	3.93 × 10^−8^	1.82 × 10^−12^	0.61
2.0	1.23 × 10^−7^	3.02 × 10^−12^	0.75
4.0	1.98 × 10^−7^	5.25 × 10^−11^	0.78
6.0	6.15 × 10^−8^	5.89 × 10^−12^	0.67

The pristine PVA/CMC blend displays the lowest maximum *σ*_ac_ value of 1.67 × 10^−8^ S cm^−1^ and a *σ*_dc_ of 9.77 × 10^−13^ S cm^−1^, confirming the insulating nature of the polymer matrix. *σ*_ac_ and *σ*_dc_ are found to be significantly increased after incorporation of Ag/MoO_3_ hybrid nanofillers. The increased conductivity may be due to the complementary contribution of conductive Ag nanoparticles and semiconducting MoO_3_ nanostructures, which provide additional charge transport pathways and localized states within the polymer network.^7,12,19^

The maximum *σ*_ac_ increases from 3.93 × 10^−8^ S cm^−1^ for 1 wt% Ag/MoO_3_ to 1.23 × 10^−7^ S cm^−1^ for 2 wt% and reaches the highest value of 1.98 × 10^−7^ S cm^−1^ for the 4 wt% Ag/MoO_3_ nanocomposite. Similarly, *σ*_dc_ increases from 1.82 × 10^−12^ S cm^−1^ for 1 wt% to 5.25 × 10^−11^ S cm^−1^ for the 4 wt% sample. The enhancement of conductivity at 4 wt% loading could be due to increased charge-carrier mobility and enhanced hopping probability between localized electronic states. The optimized concentration of nanofiller offers favourable conditions for charge transport in the composite network. This behaviour is consistent with the reduced optical band-gap energy reported for the same composition, indicating increased electronic interaction and charge-transfer capability.^12,38^

The frequency exponent (*s*) values obtained from Jonscher's power-law fitting vary from 0.60 to 0.78. The increase of *s* from 0.60 for pristine PVA/CMC to 0.78 for the composite containing 4 wt% Ag/MoO_3_ suggests an increased degree of localized charge transport and an improved interaction between the charge carriers and the heterogeneous nanofiller interfaces. The results indicate that the charge transport was dominated by hopping between localized states created *via* the nanofillers.^47,49^

Further increase of Ag/MoO_3_ loading to 6 wt% results in a reduction of maximum *σ*_ac_ and *σ*_dc_ values to 6.15 × 10^−8^ S cm^−1^ and 5.89 × 10^−12^ S cm^−1^, respectively. This decrease may be attributed to excessive nanoparticle interactions and partial aggregation, which reduce the effective interfacial area and disrupt continuous charge transport pathways.^8,47^

In brief, the electrical conductivity results showed that 4 wt% Ag/MoO_3_ is the optimum nanofiller concentration which provides the best combination of nanofiller dispersion, interfacial polarization, localized state formation and charge-carrier transport. The improved conductivity and controlled hopping behaviour suggest potential relevance for dielectric and sensing-related applications, subject to further performance evaluation.^12,48,50^

### 3.7 Dielectric properties analysis

The frequency-dependent dielectric properties of pristine PVA/CMC and Ag/MoO_3_ hybrid nanocomposite films were evaluated in terms of dielectric constant (*ε*′) and dielectric loss (*ε*″), as presented in Fig. 7. The dielectric response of polymer nanocomposites is strongly dependent on the filler concentration, the polarization and the charge-carrier mobility in the composite network.^7,19,48^

**Fig. 7:**
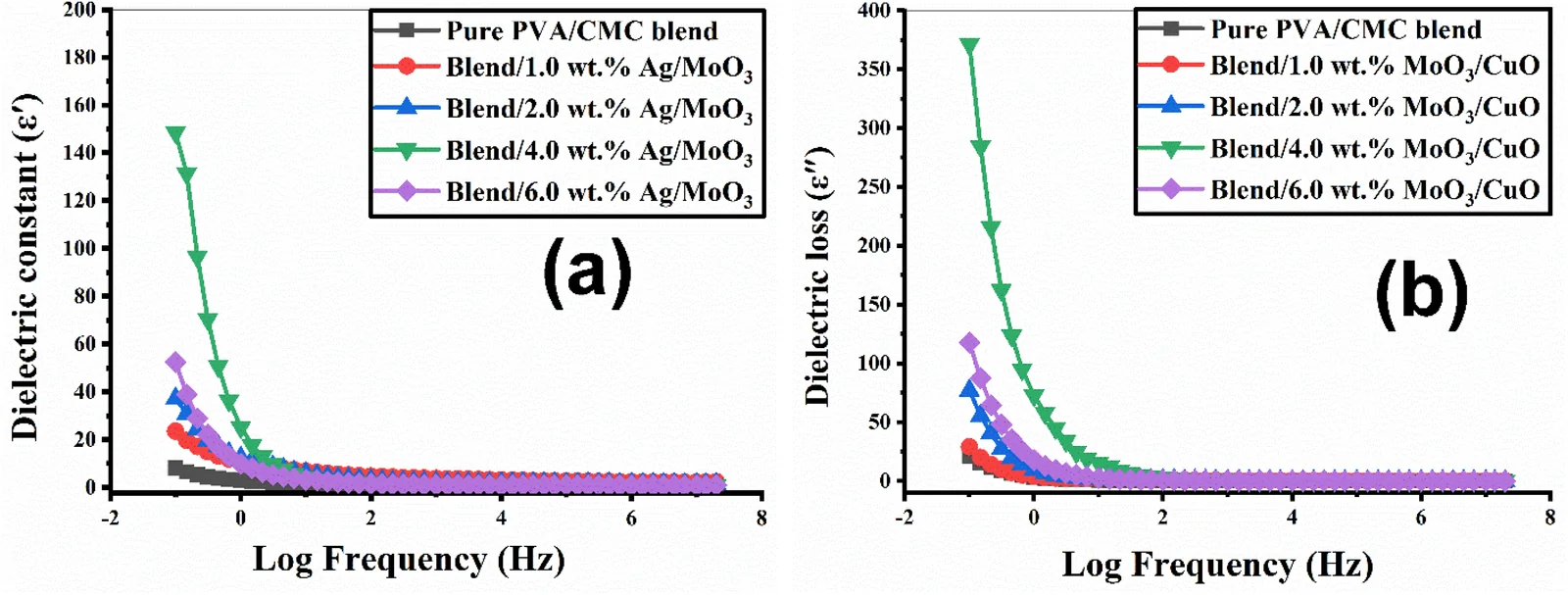
Frequency-dependent dielectric properties of PVA/CMC–Ag/MoO_3_ nanocomposite films. (a) Dielectric constant (*ε*′) and (b) dielectric loss (*ε*″).

The dielectric constant of all the samples decreases with increase in frequency due to the failure of dipoles and accumulated charge carriers to follow the rapidly alternating electrical fields at higher frequency part. At low frequency part the dielectric response is greatly influenced by different polarisation mechanisms like dipolar, interfacial and space-charge polarisations.^47,49^

The pristine PVA/CMC blend exhibits the lowest dielectric response with maximum values of *ε*′ and *ε*″ of 8.26 and 21.18, respectively. This behaviour is correlated with the low availability of mobile charge carrier and the insulating nature of the polymer matrix. The improved *ε*′ and *ε*″ values after the addition of Ag/MoO_3_ hybrid nanofillers indicate enhanced polarization within the composite system.

The maximum dielectric constant increases from 23.52 for 1 wt% Ag/MoO_3_ to 37.18 for 2 wt% and reaches the maximum value of 148.59 for 4 wt% Ag/MoO_3_ composite. Similarly, the dielectric loss maximum increases from 28.88 to 76.89 and reaches 371.19 for 4 wt% sample. The exceptional improvement at 4 wt% loading is ascribed to the presence of a large number of Ag/MoO_3_–polymer interfaces that favour the Maxwell–Wagner–Sillars interfacial polarisation and the accumulation of charges at the heterogeneous boundaries.^5,8,12^

The improved dielectric performance is ascribed to the combined contribution of conductive Ag domains and MoO_3_-induced localized states that facilitate the interfacial polarisation and charge accumulation. Ag nanoparticles act as conductive domains and MoO_3_ provides defect-related localized states for charge accumulation and polarization processes. The increased number of interfaces between nanofillers and polymer chains leads to more charge trapping regions, resulting in enhanced dielectric permittivity.^12,50^

Upon increasing the Ag/MoO_3_ loading to 6 wt%, the dielectric response decreases, with the maximum values of *ε*′ and *ε*″ reduced to 52.52 and 117.62, respectively. Such a reduction may be related to possible nanofiller aggregation at higher concentration, which could decrease the effective interfacial area and limit homogeneous charge accumulation. The dielectric behaviour of polymer nanocomposites with hybrid inorganic fillers is also concentration dependent.^47,48^

In general, the dielectric measurements confirm that Ag/MoO_3_ addition significantly modifies the polarization behaviour of the PVA/CMC matrix. The 4 wt% Ag/MoO_3_ composite exhibited the highest dielectric response due to enhanced interfacial polarization, charge accumulation, and effective filler dispersion, highlighting its suitability for dielectric and energy-storage applications.^12,48,50^

### 3.8 Electric modulus analysis

The real (*M*′) and imaginary (*M*″) modulus spectra of the polymer matricesare shown in Fig. 8. The electric modulus representation suppresses electrode polarization effects and gives information on the intrinsic relaxation processes within heterogeneous polymer systems.^51,52^

**Fig. 8:**
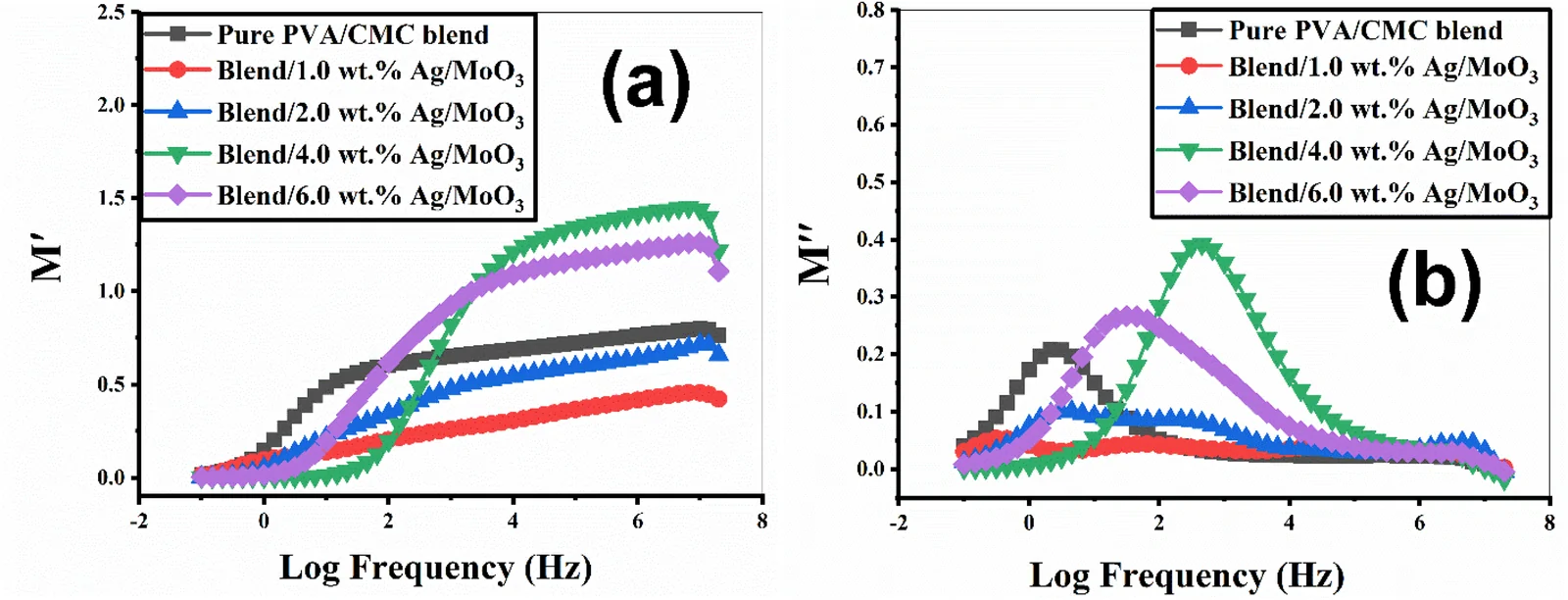
The electric modulus spectra of PVA/CMC–Ag/MoO_3_ nanocomposite films. (a) Real (*M*′) and (b) imaginary (*M*″) parts of the electric modulus.

The real modulus (*M*′) values are small at lower frequencies and increase with increasing frequency which is an indication of restricted long-range charge migration and a transition toward localized charge motion at higher frequencies. The pristine PVA/CMC blend shows a maximum value of *M*′ of 0.800, and the addition of Ag/MoO_3_ alters the modulus response due to the formation of new interfaces between the polymer and nanofiller. The 4 wt% Ag/MoO_3_ composite exhibits the highest *M*′ value (1.452) indicating improved charge localization and interfacial polarization due to the optimal dispersion of hybrid nanofillers.

The improvement of modulus response upon incorporation of Ag/MoO_3_ may be due to the combined contribution of conductive Ag nanoparticles and semiconducting MoO_3_ nanostructures. The Ag nanoparticles introduce conductive domains and MoO_3_ introduces localized states due to defects that act as charge carrier trapping sites. Such heterogeneous interfaces promote the accumulation of charge and influence the mobility of charge carriers in the polymer network.^12,48^

The imaginary modulus (*M*″) spectra present peaks of relaxation related to the charge relaxation processes of the nanocomposite films. Maximum *M*″ values increase from 0.208 for pristine PVA/CMC to 0.392 for the 4 wt% Ag/MoO_3_ composite. The relaxation maximum for the 4 wt% sample at higher frequency (log *f* ≈ 2.66) shows faster relaxation dynamics and better charge transport than the pristine polymer matrix. The relaxation time (*τ*) corresponding to the *M*″ maximum is given by:5
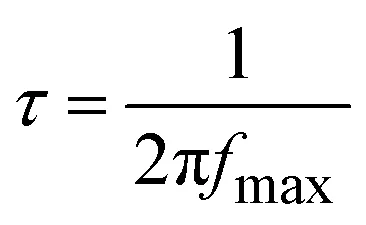
where *f*_max_ represents the frequency corresponding to the maximum value of *M*″. The calculated relaxation times show a strong dependence on Ag/MoO_3_ concentration. The relaxation time was 7.4 × 10^−2^ s for pristine PVA/CMC mayrix and increased to 5.1 × 10^−1^ s for the 1 wt% Ag/MoO_3_ composite. With further nanofiller incorporation, the relaxation time decreased to 3.5 × 10^−2^ s, 3.5 × 10^−4^ s, and 5.1 × 10^−3^ s for the 2, 4, and 6 wt% Ag/MoO_3_ samples, respectively. The shortest relaxation time observed for the 4 wt% Ag/MoO_3_ nanocomposite indicates faster charge relaxation and improved carrier mobility due to enhanced interfacial interactions and localized charge transport.^12,48,51,52^ The increase in relaxation time at higher loading (6 wt%) may be related to nanoparticle aggregation and reduced efficiency of charge transport pathways caused by excessive filler concentration.^8,47^

The shift in the position and intensity of the relaxation peaks confirm the fact that addition of Ag/MoO_3_ changes the charge relaxation behaviour of the PVA/CMC matrix.^51,52^ The broad *M*″ peaks imply a distribution of relaxation times characteristic of non-Debye relaxation from the coupling of polymer segmental dynamics, interfacial polarisation and localized charge hopping in the heterogeneous nanocomposite structure.^5,48^

A higher Ag/MoO_3_ loading (6 wt%) led to a lower modulus response than 4 wt%, with maximum *M*′ and *M*″ values of 1.262 and 0.267, respectively. This reduction may be attributed to partial aggregation of the nanofiller resulting in a reduction in the effective interfacial area and consequently affecting the uniform distribution of charges in the polymer matrix.^8,47^

In conclusion, electric modulus analysis verifies the significant effect of Ag/MoO_3_ hybrid nanofillers on the relaxation behaviour and charge transport mechanism of PVA/CMC matrices. The optimum balance between interfacial polarization, localized charge trapping and carrier mobility is obtained for the 4 wt% Ag/MoO_3_ composition, which is in good agreement with the enhanced electrical and dielectric responses observed in this study.^12,48,50^

### 3.9 Impedance spectroscopy

Impedance analysis was used to investigate the electrical response and relaxation behaviour of PVA/CMC and Ag/MoO_3_/PVA/CMC nanocomposite films. The values of real (*Z*′) and imaginary (*Z*″) part of impedance were presented in Fig. 9. The gradual decrease of *Z*′ with frequency indicates improved charge transport at higher frequencies. The pristine PVA/CMC film exhibits higher impedance, whereas Ag/MoO_3_ incorporation reduces impedance by facilitating charge migration and creating conductive pathways within the composite structure.^12,48^

**Fig. 9:**
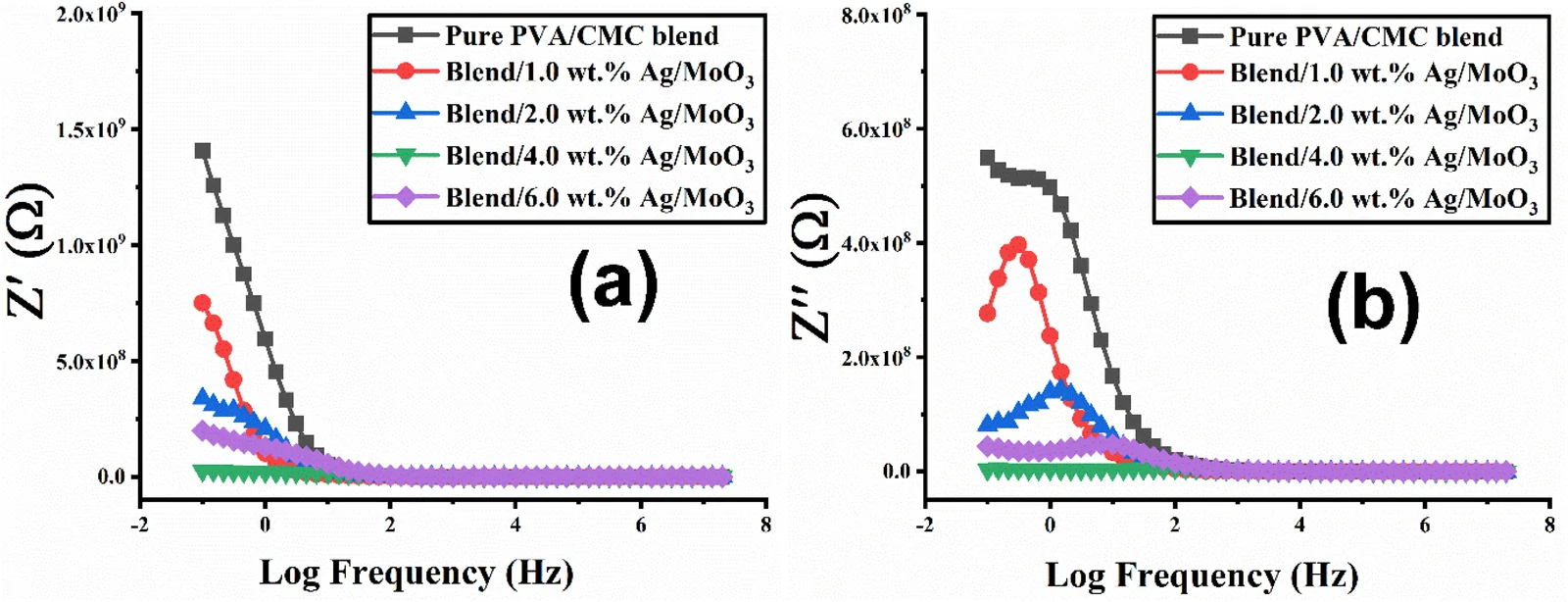
(a) Real (*Z*′) and (b) imaginary (*Z*″) parts of impedance of PVA/CMC–Ag/MoO_3_ nanocomposite films.

The reduction in impedance after the addition of Ag/MoO_3_ can be explained by the combined contribution of metallic Ag nanoparticles and semiconducting MoO_3_ nanostructures. Metallic Ag nanoparticles provide conducting domains, while MoO_3_ creates localized electronic states for hopping of charge within the polymer matrix. Also, the heterogeneous interfaces between Ag/MoO_3_ and PVA/CMC chains facilitated interfacial polarization and charge accumulation, and thus contributed to the improved electrical response.^8,12,48^

The increase in impedance at low frequency part is mainly due to the accumulation of charges at the interfaces electrode-sample and filler–polymer, giving rise to the interfacial polarisation effects of the electrode and the Maxwell–Wagner–Sillars.^48,50^ The slow polarisation processes are limited at higher frequencies due to the charge carriers inability to follow the rapidly changing electric field which causes a slow decrease in impedance.

The spectra of imaginary impedance (*Z*″) gives information about dielectric relaxation and energy dissipation processes in the nanocomposite films. The broad relaxation response observed in the samples suggests a distribution of relaxation time rather than a single relaxation process. This behaviour is typical of heterogeneous polymer nanocomposites, where the electrical response was related to the combined contributions of polymer segmental motion, interfacial polarisation and localized charge hopping.^51,52^

The Ag/MoO_3_-containing films show lower *Z*″ compared with pristine PVA/CMC, confirming the increased charge mobility and the modified relaxation behaviour upon nanofiller incorporation. The most improved response is observed in the 4 wt% Ag/MoO_3_ composite, which exhibits the highest ionic conductivity and dielectric response, owing to the best dispersion of the nanofillers and efficient construction of the interfacial charge transport pathways.^52,53^

The impedance analysis reveals that the addition of Ag/MoO_3_ enhances the electrical properties of the PVA/CMC matrix by reducing impedance, increasing interfacial polarisation, and facilitating charge transport. The maximum response at 4 wt% Ag/MoO_3_ is consistent with AC conductivity, dielectric and modulus results. This highlights the role of filler concentration in tuning properties of nanocomposites.^12,48,50^

### 3.10 Nyquist plot and equivalent circuit analysis

Nyquist plots of the prepared films are presented in Fig. 10, and the corresponding equivalent circuit parameters are summarized in Table 3. The impedance data were fitted using a circuit model containing bulk resistance (*R*_b_) and a constant phase element (CPE). For Ag/MoO_3_-modified samples, an additional CPE was incorporated to account for interfacial polarization arising from the polymer–nanofiller interface. The CPE impedance relation is expressed as:^54^6
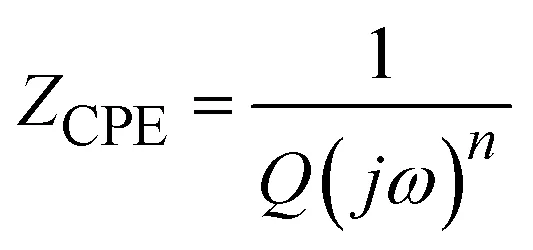
where *Q* is the CPE parameter, *j* represents the imaginary unit, *ω* = 2π*f*, and *n* is the CPE is the exponent that describes the deviation from ideal capacitive behaviour. An *n* value close to 1 is characteristic of ideal capacitive behaviour whereas lower *n* values are indicative of non-ideal capacitive behaviour related with structural heterogeneity and distributed relaxation processes.^53,54^

**Fig. 10:**
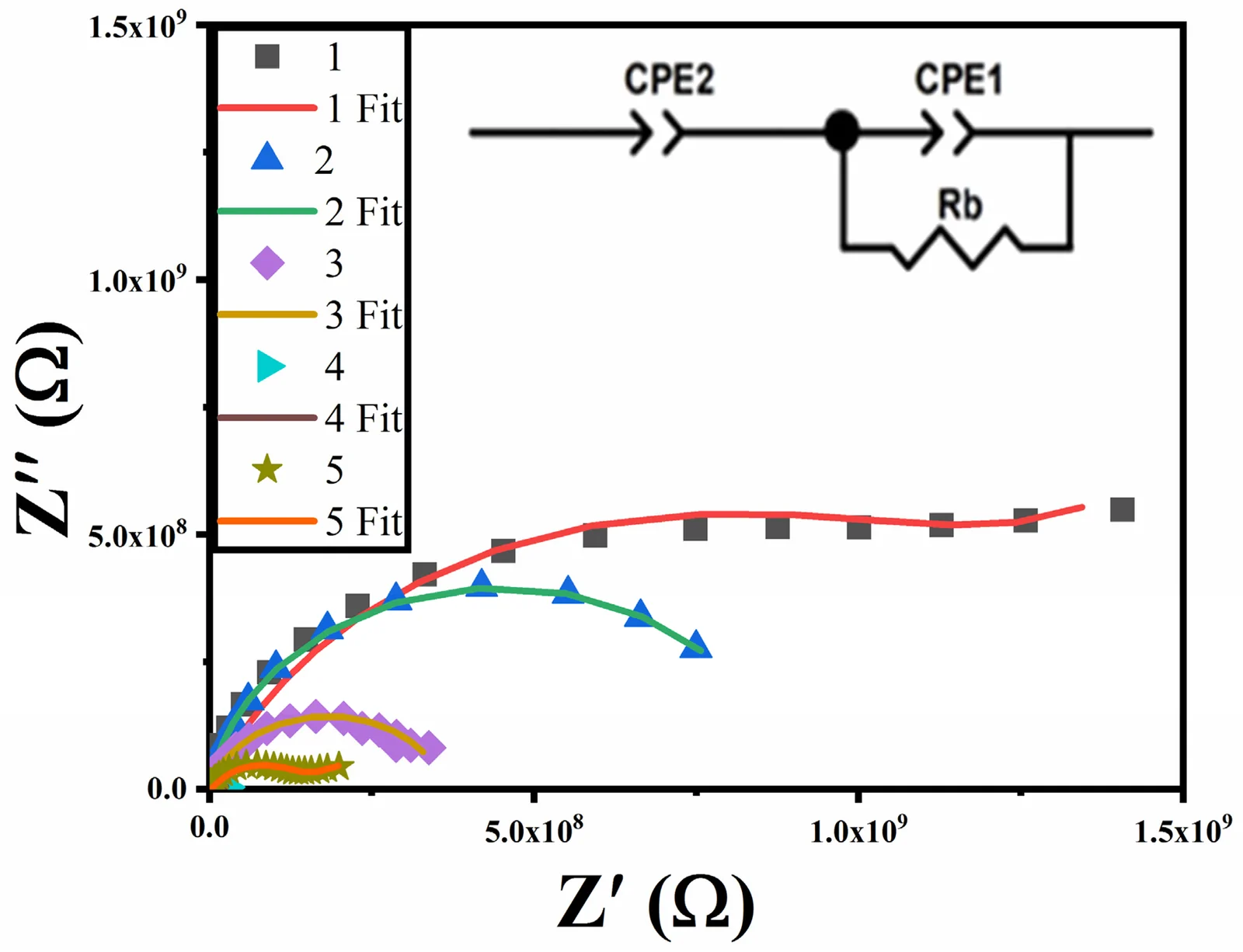
Nyquist plots (*Z*″ *vs. Z*′) and equivalent circuit models of PVA/CMC–Ag/MoO_3_ nanocomposite films: (1) pristine PVA/CMC matrix, (2) 1 wt% Ag/MoO_3_ nanocomposite, (3) 2 wt% Ag/MoO_3_ nanocomposite, (4) 4 wt% Ag/MoO_3_ nanocomposite, and (5) 6 wt% Ag/MoO_3_ nanocomposite.

**Table 3:** Equivalent circuit fitting parameters of pristine PVA/CMC and Ag/MoO_3_-modified PVA/CMC nanocomposite films

Concentrations of Ag/MoO_3_ (wt%)	Fitting parameters				
	*R*_b_ (Ω)	*Q*_1_ (F)	*n*_1_	*Q*_2_ (F)	*n*_2_
0.0	1.14 × 10^9^	2.23 × 10^−10^	0.92	—	—
1.0	8.93 × 10^8^	6.51 × 10^−10^	0.81	—	—
2.0	3.65 × 10^8^	9.86 × 10^−10^	0.85	2.79 × 10^−9^	0.59
4.0	1.56 × 10^7^	6.83 × 10^−10^	0.73	9.08 × 10^−8^	0.33
6.0	1.12 × 10^8^	2.90 × 10^−10^	0.75	1.18 × 10^−8^	0.30

The Nyquist plots show depressed semicircular arcs instead of ideal semicircles indicating non-ideal capacitive behaviour and a distribution of relaxation time in the nanocomposite films. Such deviations from the ideal behaviour are often observed in heterogeneous polymer nanocomposites due to structural disorder, interfacial polarization, defects and differences in conductivity between the polymer matrix and inorganic nanophases.^48,53,54^

The values obtained for the fitted bulk resistance (*R*_b_) show a strong dependence on the Ag/MoO_3_ concentration. The pristine PVA/CMC blend shows the highest resistance value of 1.14 × 10^9^ Ω, indicating the low charge transport capacity of the polymer matrix. The *R*_b_ decreases progressively to 8.93 × 10^8^, 3.65 × 10^8^, 1.56 × 10^7^, and 1.12 × 10^8^ Ω after incorporation of Ag/MoO_3_ hybrid nanofillers for 1, 2 and 4 wt% Ag/MoO_3_ samples respectively. The significant decrease in resistance shows that the formation of additional charge-transport pathways leads to an improvement in electrical conductivity.^53,55^

The improved electrical response may arise from the complementary contributions of metallic Ag nanoparticles and semiconducting MoO_3_ nanostructures. The Ag nanoparticles act as conductive regions and MoO_3_ provides defect related localized states which can support charge hopping and carrier migration within the polymer matrix. Moreover, the increased Ag/MoO_3_–polymer interfaces provide the Maxwell–Wagner–Sillars polarization to enhance the charge accumulation and electrical response.^12,48,50^

For the highest filler concentration (6 wt% Ag/MoO_3_), the bulk resistance increases slightly to 1.12 × 10^8^ Ω. This increase compared with the 4 wt% sample could be due to possible partial nanofiller aggregation or reduced interfacial effectiveness at higher loading, which could disrupt the efficient charge transport pathways. Similar concentration-dependent behaviour has been reported in polymer nanocomposites where excessive filler loading decreases the efficiency of charge migration due to agglomeration effects.^8,47^

The CPE parameters also support heterogeneous dielectric nature of nanocomposite system. The values of the CPE exponent (*n*) lie between 0.73 and 0.92 which indicate deviation from the ideal capacitive behaviour and existence of non-Debye relaxation processes. The decrease of *n* values after addition of nanofiller is attributed to the increase of structural disorder and wider distribution of relaxation time caused by the incorporation of Ag/MoO_3_ nanophases.^48,53,54^

The additional CPE contribution noticed for the three higher Ag/MoO_3_ concentrations (2, 4 and 6 wt%) suggests the presence of extra interfacial relaxation processes due to the heterogeneous Ag/MoO_3_–polymer interfaces. These interfaces are active sites for charge accumulation and control the dielectric and impedance responses of the nanocomposite films.^12,50,53^ Thus, we added another CPE element for these samples to describe the interfacial polarization corresponding to the Ag/MoO_3_–PVA/CMC interfaces. The bulk resistance (*R*_b_) is the resistance of the composite matrix to charge transport, while the CPE elements describe the non-ideal capacitive behaviour due to structural heterogeneity, charge accumulation and distributed relaxation processes.

Overall, the equivalent circuit analysis supports the concentration dependent electrical response and highlights the role of Ag/MoO_3_–polymer interfaces in controlling the resistance and relaxation behaviour.

### 3.11 Antibacterial activity analysis

The antibacterial activity of pristine PVA/CMC and Ag/MoO_3_-modified PVA/CMC nanocomposite films was tested by inhibition zone method against bacteria *Staphylococcus* spp. (Gram-positive) and *Klebsiella pneumoniae* (Gram-negative). Antibacterial results are shown in Fig. 11 and Table 4.

**Fig. 11:**
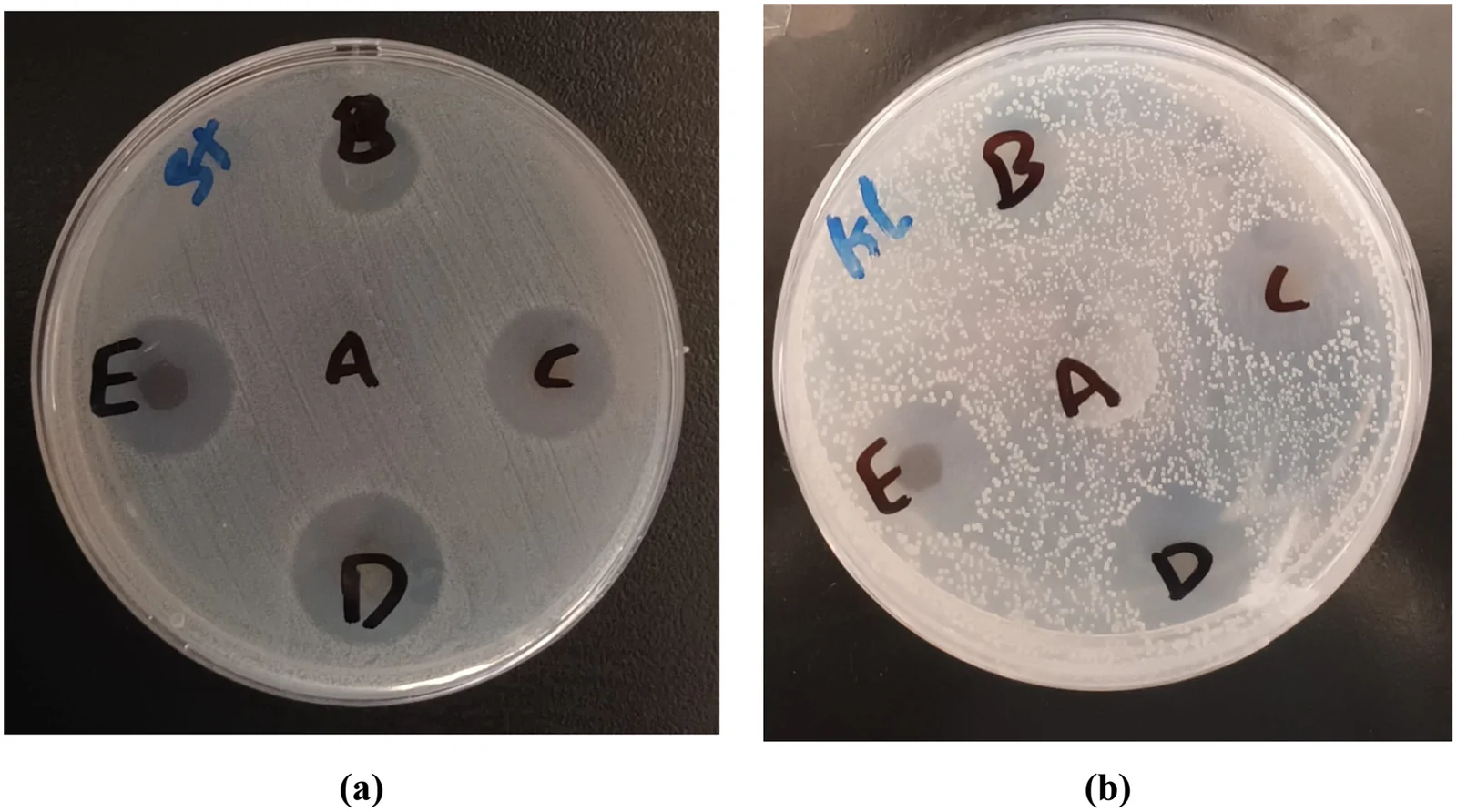
Antibacterial activity of PVA/CMC–Ag/MoO_3_ nanocomposites against bacterial strains: (a) *Staphylococcus* spp. (Gram-positive) and (b) *Klebsiella pneumoniae* (Gram-negative), demonstrating the concentration-dependent inhibition effect of Ag/MoO_3_ hybrid nanofiller incorporation.

**Table 4:** Antimicrobial performance of PVA/CMC–Ag/MoO_3_ nanocomposite films against *Staphylococcus aureus* (ATCC 29213) and *Klebsiella pneumoniae* (ATCC 43816) evaluated by inhibition zone diameter (mm). Values are expressed as mean ± standard deviation (*n* = 3)

Concentration of Ag/MoO_3_ (wt%)	*Staphylococcus* spp. inhibition zone (mm)	*Klebsiella pneumoniae* inhibition zone (mm)
0.0	0 ± 0	0 ± 0
1.0	13 ± 1	15 ± 1
2.0	24 ± 1	28 ± 1
4.0	36 ± 1	34 ± 1
6.0	33 ± 1	30 ± 1

No antibacterial activity was observed for the pristine PVA/CMC blend as evidenced by the 0 mm inhibition zones for both bacterial strains. This behaviour may be explained by the lack of active antimicrobial components in the polymer matrix, indicating that the PVA/CMC blend mainly provides a biocompatible supporting network rather than active antibacterial functionality. Similar observations have been reported for neat PVA/CMC films, where antibacterial activity was significantly enhanced only after incorporation of antimicrobial nanofillers such as silver-containing nanoparticles.^19,41^

On incorporation of Ag/MoO_3_ hybrid nanofillers concentration-dependent antibacterial response was observed. The inhibition zones obtained with 1.0 wt% Ag/MoO_3_ loading were 13 mm and 15 mm against *Staphylococcus* spp. and *Klebsiella pneumoniae*, respectively, demonstrating that a low concentration of hybrid nanofillers can impart significant antibacterial activity. The Ag/MoO_3_ concentration was raised to 2.0 wt% to enhance the inhibition zones to 24 mm for *Staphylococcus* spp. and 28 mm for *Klebsiella pneumoniae*. The improved antibacterial activity may be related to the enhanced exposure of the active surfaces of nanofillers and the stronger interaction between the bacterial cells and the Ag/MoO_3_ nanoparticles.^20^

The nanocomposite with 4.0 wt% Ag/MoO_3_ exhibited maximum antibacterial activity with inhibition zones of 36 mm and 34 mm for *Staphylococcus* spp. and *Klebsiella pneumoniae*, respectively. The improved antimicrobial performance at this concentration is consistent with the optimum structural and electrical properties obtained in this study. The better distribution of Ag/MoO_3_ nanophases results in a larger effective surface area and more interfaces between polymer and nanofiller, which favours the release of antibacterial active sites and their interaction with microbial cells.

The antibacterial mechanism of Ag/MoO_3_ modified nanocomposites can be ascribed to the complementary contributions of silver nanoparticles and molybdenum trioxide nanostructures. Silver nanoparticles are known to show antibacterial activity through several mechanisms such as interaction with bacterial cell membrane, disruption of membrane integrity, generation of reactive oxygen species (ROS) and interaction with intracellular biomolecules.^2,18,55^ The Ag species may enter the bacterial cell and interrupt important metabolic processes, thus inhibiting the growth of the bacteria. Meanwhile, the semiconducting nature of MoO_3_, the oxygen-defect-related active sites, and its ability to participate in oxidative stress-related antibacterial pathways contribute. Thus, Ag and MoO_3_ together give complementary antibacterial effects by improving surface activity and encouraging microbial inactivation.^41,55^

The stronger response against *Klebsiella pneumoniae* at lower Ag/MoO_3_ concentrations could be associated with differences in the bacterial surface structure and interaction pathways between Gram-negative and Gram-positive bacteria. *Klebsiella pneumoniae* lipopolysaccharides on the outer membrane modulate nanoparticle adhesion, permeability and susceptibility to metal-based antimicrobial agents.^18,55^ However, at the highest tested loading, both bacterial strains showed significant inhibition confirming the broad spectrum antibacterial capability of the hybrid nanocomposite system.

When the Ag/MoO_3_ content reached 6.0 wt%, antibacterial activity decreased slightly; the inhibition zones shrank to 33 mm for *Staphylococcus* spp. and 30 mm for *Klebsiella pneumoniae*. The reduction may be related to possible nanofiller aggregation at higher concentration that may reduce the available active surface area and limit effective interaction between nanoparticles and bacterial cells. Similar concentration dependent behaviour has been observed in polymer nanocomposites based on metal nanoparticles where too much filler loading can decrease antimicrobial efficacy due to particle clustering.^14,41^

To summarise, the antibacterial results demonstrate that the addition of Ag/MoO_3_ effectively changes the inert PVA/CMC matrix into an active antibacterial nanocomposite material. The 4 wt% Ag/MoO_3_ composition provides the optimum antibacterial performance together with improved optical, and dielectric properties, highlighting its potential for antimicrobial coatings, functional packaging materials, and flexible polymer-based applications.^12,19,20^

## 4 Conclusion

Ag/MoO_3_ hybrid nanofiller-reinforced PVA/CMC nanocomposite films were successfully prepared *via* a solution-casting method, followed by comprehensive evaluation of their structural, optical, electrical, impedance, and antibacterial characteristics. XRD and FTIR analyses confirmed the successful addition of Ag and MoO_3_ phases and the presence of effective polymer–nanofiller interactions. The addition of 4 wt% Ag/MoO_3_ decreased the crystallinity of the PVA/CMC blend from 36.6% to 19.4%, which suggests an increase in structural disorder and interfacial interactions.

Incorporation of Ag/MoO_3_ improved the optical properties considerably. The indirect and direct optical band-gaps at 4 wt% loading reduced from 4.78 to 2.52 eV and 5.22 to 3.14 eV, respectively, due to the formation of localized electronic states and modified electronic structure.

The electrical and dielectric properties showed strong concentration dependence. The 4 wt% Ag/MoO_3_ nanocomposite exhibited the highest AC conductivity (1.98 × 10^−7^ S cm^−1^), DC conductivity (5.25 × 10^−11^ S cm^−1^), dielectric constant (148.59), and dielectric loss (371.19). Impedance analysis confirmed improved charge transport, with bulk resistance reduced from 1.14 × 10^9^ Ω for pristine PVA/CMC to 1.56 × 10^7^ Ω for the 4 wt% composite. The non-Debye relaxation behaviour was assigned to heterogeneous Ag/MoO_3_–polymer interfaces and interfacial polarization.

The antibacterial assessment showed a concentration-dependent activity, with the 4 wt% Ag/MoO_3_ film having the highest inhibition zones of 36 mm against *Staphylococcus* spp. and 34 mm against *Klebsiella pneumoniae*.

Overall, the optimum balance of structural, optical, electrical, dielectric and antibacterial properties was achieved for the 4 wt% Ag/MoO_3_ nanocomposite, which was attributed to the combined contributions of the Ag and MoO_3_ phases. The obtained results demonstrate that Ag/MoO_3_-modified PVA/CMC nanocomposites are promising candidates for electronic devices, dielectric components, antimicrobial coatings, and functional packaging applications. However, further investigations are still required for practical implementation, such as mechanical flexibility, long-term stability, and device-level performance evaluations.

## Author contributions

G. M. A., M. ALSaeedy, and Z. M. A. conceptualized the study. The investigation and methodology were performed by G. M. A., M. ALSaeedy, M. M. B., and Z. M. A. The original manuscript draft was prepared by G. M. A., M. ALSaeedy, M. M. B., and Z. M. A. Review and editing of the manuscript were conducted by G. M. A., M. ALSaeedy, R. U., M. M. B., Z. M. A., A. Z. A., S. M. A., and M. K. G. All authors reviewed and approved the final version of the manuscript for publication.

## Conflicts of interest

The authors declare that they have no known competing financial interests or personal relationships that could have appeared to influence the work reported in this paper.

## Data Availability

The data supporting the in findings of this study are available within the article. Additional experimental data (including synthesis and biological assay results) are available from the corresponding author upon reasonable request.
